# Supplementary material for: Next generation plasma proteome profiling to monitor health and disease
Source: Nat Commun. 2021 May 3;12:2493. doi: 10.1038/s41467-021-22767-z (PMC8093230; doi:10.1038/s41467-021-22767-z)
Supplement: Supplementary file 1 — Supplementary Information [file 41467_2021_22767_MOESM1_ESM.pdf]

Supplementary Information for

Zhong et al. Next generation plasma proteome profiling to monitor health and disease

The PDF file includes:

Supplementary Figure 1. Intensity sample normalization and quality assessment.
Supplementary Figure 2. Comparisons of intra-/inter-individual variations between PEA with NGS and qPCR measurements.
Supplementary Figure 3. Plasma proteins associated with obesity groups in type 2 diabetes cohort.
Supplementary Figure 4. Fasting glucose associated proteins in different metformin response groups.
Supplementary Figure 5. Expression patterns of proteins associated with metformin treatment response at baseline.

Supplementary Note 1. STROBE statement
Supplementary Note 2. Clinical study protocol of the wellness profile
Supplementary Note 3. Clinical study protocol of the T2D profile

Supplementary figures

Supplementary Figure 1

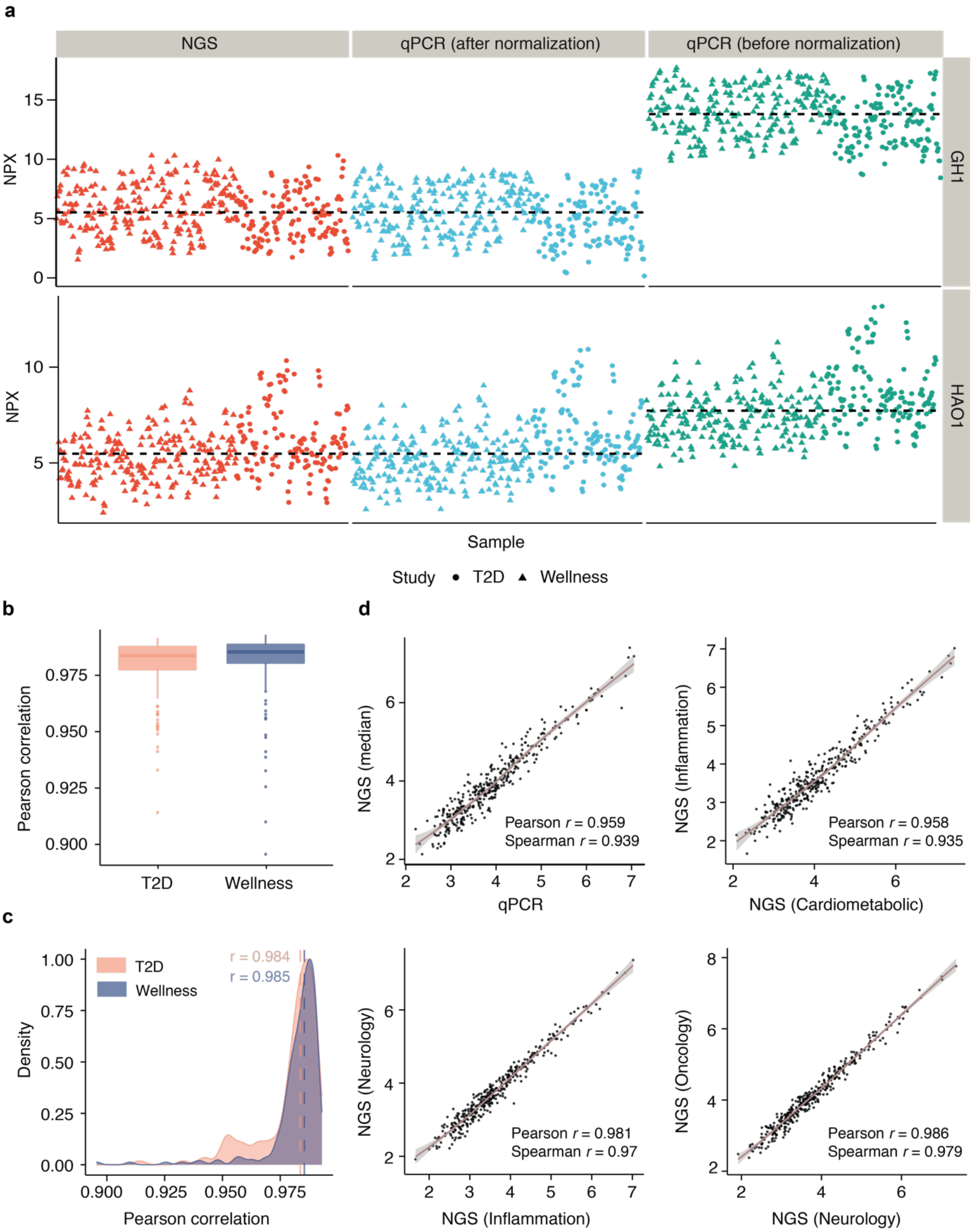

Supplementary Figure 1. Intensity sample normalization and quality assessment. (a) Two examples of the levels of proteins before and after normalization in NGS and qPCR platforms. (b) The distribution of pairwise Pearson correlation between NGS and qPCR measurements

for each sample in wellness and T2D cohorts, respectively. Box plots show medians and the 25th and 75th percentiles, whiskers show the largest and smallest values (n = 372 samples). (c) A combined boxplot and density plot showing the distribution of the sample-wise correlation value between NGS and qPCR measurements for each sample in wellness and T2D cohorts, respectively. (d) Scatter plots showing pairwise correlation between data from different panels and from different platforms for the IL6 protein. Source data are provided as a Source Data file.

Supplementary Figure 2

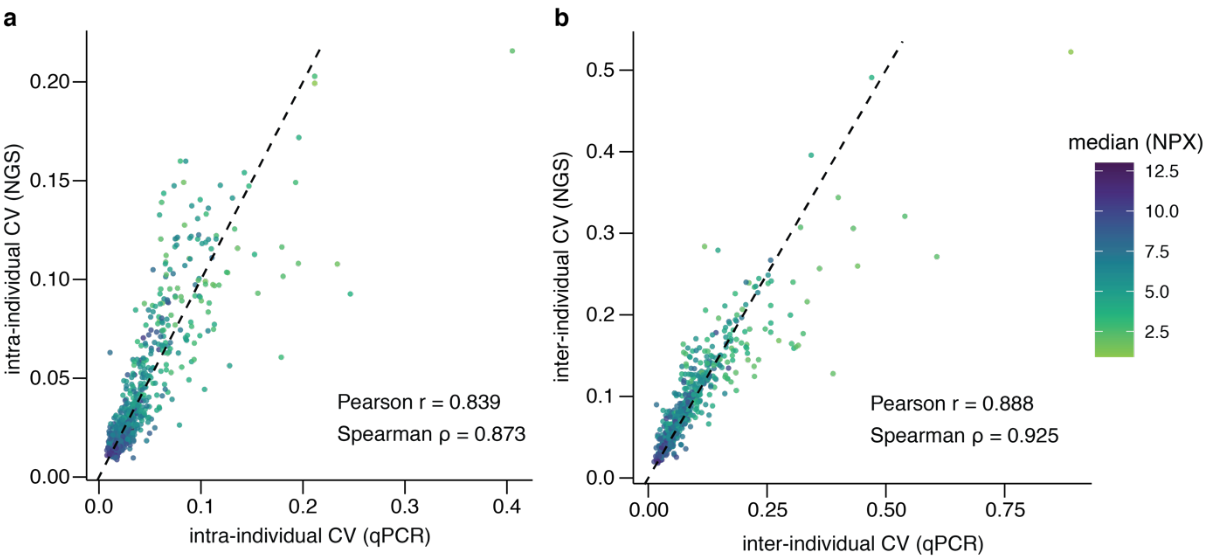

Supplementary Figure 2. Comparisons of intra-/inter-individual variations between PEA with NGS and qPCR measurements. (a) A scatter plot showing pairwise correlation of mean coefficient of variation (CV) between NGS and qPCR platforms for each protein within each individual across three visits in the wellness study. (b) A scatter plot showing pairwise correlation of mean CV between NGS and qPCR platforms for each protein within each visit across all 76 analyzed individuals in the wellness study. Source data are provided as a Source Data file.

Supplementary Figure 3

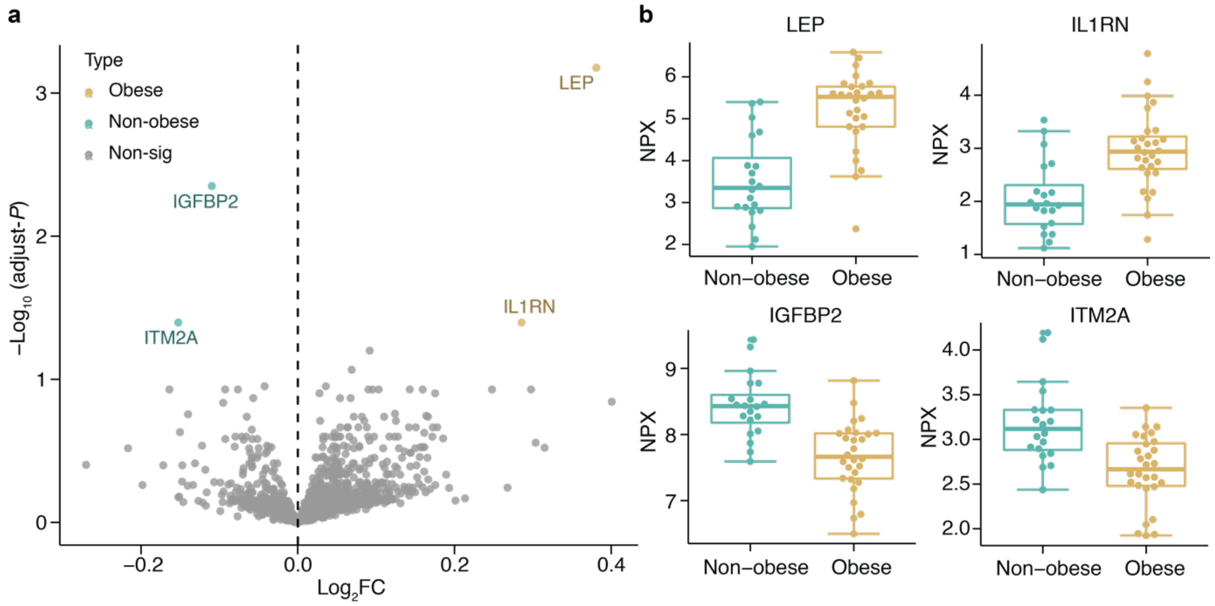

Supplementary Figure 3. Plasma proteins associated with obesity groups in type 2 diabetes cohort. (a) A volcano plot showing the differentially expressed proteins at baseline in the non-obesity and obesity groups in the T2D cohort. The X-axis represents $\log_2$ fold-change (FC) and the Y-axis represents $\log_{10}$ (adjusted *P*-values). Differentially expressed proteins were defined as proteins with adjusted *P*-values < 0.05 (two-way balanced ANOVA for obesity with gender as covariate). Multiple test corrections have been applied for *P*-values using Benjamini and Hochberg method. (b) Four examples of the up-regulated and down-regulated proteins in the obese individuals compared to the non-obese individuals in the T2D cohort. Box plots show medians and the 25th and 75th percentiles, whiskers show the largest and smallest values (n = 48 samples). Source data are provided as a Source Data file.

Supplementary Figure 4

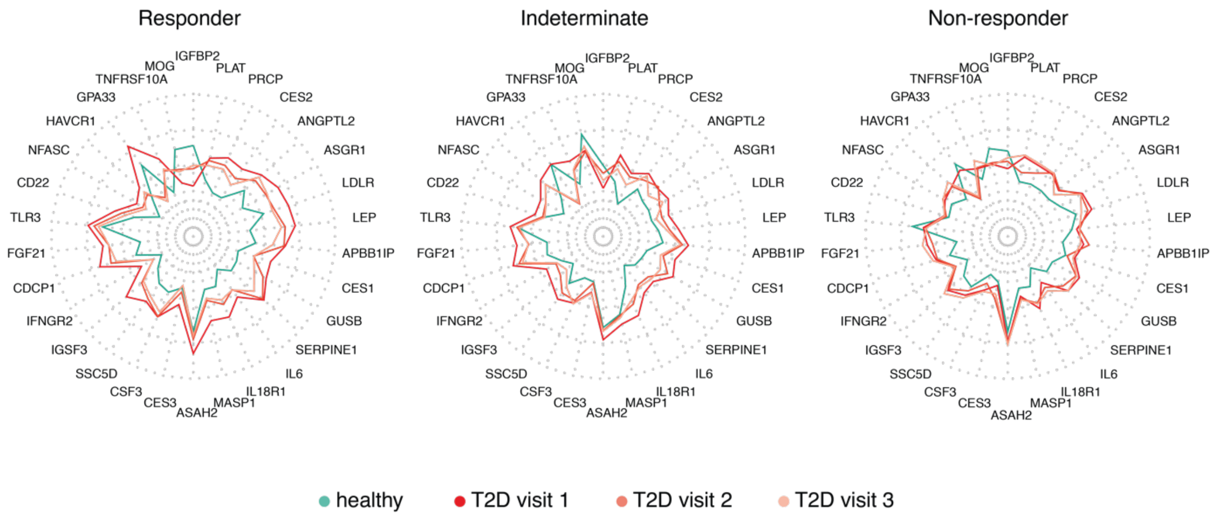

Supplementary Figure 4. Fasting glucose associated proteins in different metformin response groups. Radar plots showing average levels of top 50 fasting glucose associated proteins in the three different metformin response groups at each timepoint during the T2D study, and in the wellness cohort as median of the three visits.

Supplementary Figure 5

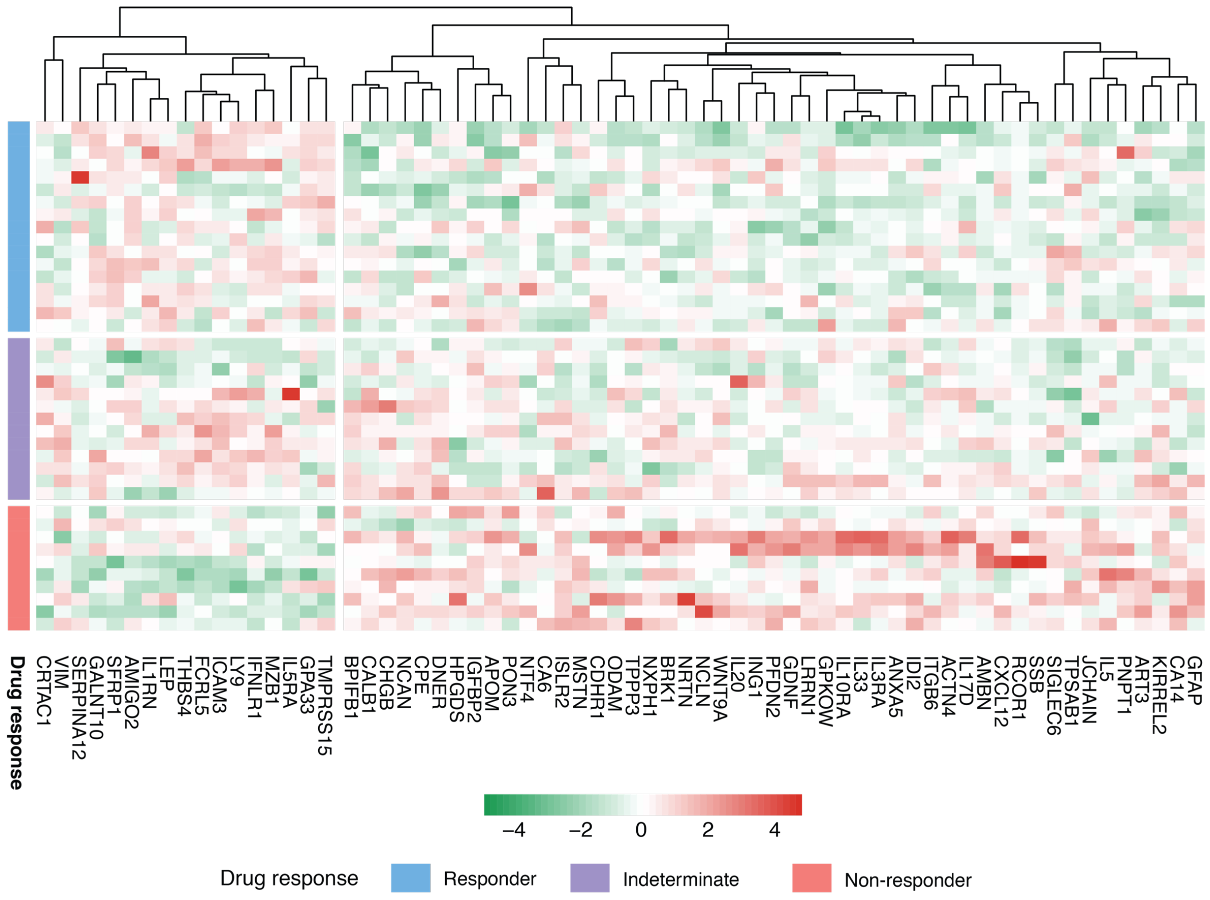

Supplementary Figure 5. Expression patterns of proteins associated with metformin treatment response at baseline. A heatmap showing the expression patterns of 66 significant proteins ($P < 0.05$) associated with metformin response at baseline in all 40 subjects with metformin treatment (mixed-effect modeling analysis for drug response with gender as covariate).

Supplementary Note 1

STROBE Statement—Checklist of items that should be included in reports of ***cohort studies***

	Item No	Items (Recommendation)
**Title and abstract**	1	**(*a*) Indicate the study's design with a commonly used term in the title or the abstract**<br><br>Next generation plasma proteome profiling to monitor health and disease
		**(*b*) Provide in the abstract an informative and balanced summary of what was done and what was found**<br><br>We describe a novel approach for plasma profiling based on proximity extension assay combined with next generation sequencing. First, the variability of plasma profiles between and within healthy individuals was analyzed in a longitudinal wellness study, including the influence of genetic variations on plasma levels. Second, patients newly diagnosed with type 2 diabetes were followed before and during therapeutic intervention using plasma proteome profiling. The studies show that healthy individuals have a unique and stable proteome profile and that a panel of proteins can potentially be used for early diagnosis of diabetes.
**Introduction**		
Background/rationale	2	**Explain the scientific background and rationale for the investigation being reported**<br><br>The objective is to probe the circulating plasma proteome of individuals with sensitive and specific assays that can allow massive sample throughput.
Objectives	3	**State specific objectives, including any prespecified hypotheses**<br><br>Next generation plasma profiling - Proximity Extension Assay (PEA) with massive parallel sequencing
**Methods**		
Study design	4	**Present key elements of study design early in the paper**<br><br>The Swedish SciLifeLab SCAPIS Wellness Profiling (S3WP) program is non-interventional with the aim to collect longitudinal clinical and molecular data in a community-based cohort<br><br>The T2D study is an extension of the S3WP study with the aim to perform molecular phenotyping of T2D before and after diabetes treatment
Setting	5	**Describe the setting, locations, and relevant dates, including periods of recruitment, exposure, follow-up, and data collection**<br><br>The S3WP program (wellness) consists of 101 healthy individuals recruited from the Swedish CArdioPulmonary bioImage Study (SCAPIS), which is a prospective observational study with 30,154 individuals enrolled at ages between 50 and 64 years from a random sampling of the general Swedish population, from October 2015 to January 2018. Examinations in combination of sample collection (blood, urine and feces) were performed every third month (± 2 weeks) in the first year and approximately a 6-month interval in the second year.

		The T2D study consists of 52 subjects at age between 50 and 65 years with no history of diabetes and diagnosed during population-based screening examinations were enrolled from the Sahlgrenska University Hospital, Gothenburg, from March 2016 to June 2018. Examinations were performed at baseline and after 1 and 3 months of guideline-based diabetes treatment according to first-line therapy with lifestyle change including weight management and physical activity, with or without metformin as judged by the treating physician.
Participants	6	**(*a*) Give the eligibility criteria, and the sources and methods of selection of participants. Describe methods of follow-up**<br><br>The inclusion criteria for the S3WP program was the willingness to allow extensive sampling and physical examination every three months for two years and the exclusion criteria in the S3WP study included: 1) previously received health care for myocardial infarction, stroke, peripheral artery disease or diabetes, 2) presence of any clinically significant disease which, in the opinion of the investigator, may interfere with the results or the subject´s ability to participate in the study, 3) any major surgical procedure or trauma within 4 weeks of the first study visit, or 4) medication for hypertension or hyperlipidemia.<br><br>The diagnosis of diabetes in the T2D study was based on the Swedish standard, corresponding to the American Diabetes Association standards (1): A fasting p-glucose $\geqslant$7.0 mmol/L or a 2-hour oral glucose tolerance tests (OGTT) p-glucose $\geqslant$11.1 mmol/L ($\geqslant$12.2 mmol/L when measured capillary). Subjects who met diabetes criteria were scheduled for a second glucose measurement on a separate occasion and enrolled if diabetes diagnosis was confirmed.
Variables	7	**Clearly define all outcomes, exposures, predictors, potential confounders, and effect modifiers. Give diagnostic criteria, if applicable**<br><br>Not applicable.
Data sources/ measurement	8*	**For each variable of interest, give sources of data and details of methods of assessment (measurement). Describe comparability of assessment methods if there is more than one group**<br><br>Examinations and questionnaires:<br>All visits in the wellness study and the T2D study were performed using the same protocol. All subjects were fasting overnight (at least 8 hours) before the visits. Physical examinations included height, body weight, waist- and hip circumference, body fat using bioelectrical impedance (Tanita MC-780MA) and blood pressure (Omron P10). The body mass index (BMI) was calculated by dividing the weight (kg) by the square of the height (m). A selection of questions from the initial SCAPIS questionnaire was repeated to note any changes in health and lifestyle factors between each visit.<br><br>Clinical chemistry and hematology measurements:<br>Clinical chemistry and hematology measurements included fasting glucose, haemoglobin A1c (HbA1c), triglycerides (TG), total cholesterol, low-density lipoprotein (LDL), high-density lipoprotein (HDL), apolipoprotein A1 (ApoA1), apolipoprotein B (ApoB), ApoA1/B ratio, creatinine, high sensitive C-reactive protein (hsCRP), alanine aminotransferase (ALAT), gamma-glutamyltransferase (GGT), urate, cystatin C, troponin T (TNT), N-terminal pro-brain natriuretic peptide (NT-proBNP), haemoglobin (Hb), white blood cell count (WBC), red blood cell

		count (RBC) and platelet count. In addition, insulin and C-peptide was measured in the diabetes group and the homeostatic model assessment of insulin resistance (HOMA-IR) was calculated according to the formula: fasting insulin (mU/L) x fasting glucose (mmol/L) / 22.5. In total, a variety of 33 clinical chemistry parameters were included in the study.
Bias	9	**Describe any efforts to address potential sources of bias**<br><br>Random sampling from the general population, sex-balanced, within a certain age range, samples from different visits were also completely randomized across different plates
Study size	10	**Explain how the study size was arrived at**<br><br>The inclusion criteria for the S3WP program was the willingness to allow extensive sampling and physical examination. No statistical methods were applied for sample size determination.
Quantitative variables	11	**Explain how quantitative variables were handled in the analyses. If applicable, describe which groupings were chosen and why**<br><br>Statistical analysis and multiple testing were conducted to analyze the associations between different variables.
Statistical methods	12	**(*a*) Describe all statistical methods, including those used to control for confounding**<br><br>Mixed-effect modelling, analysis of variance (ANOVA), principle component analysis (PCA)
		**(*b*) Describe any methods used to examine subgroups and interactions**<br><br>mixed-effect modelling and ANOVA
		**(*c*) Explain how missing data were addressed**<br><br>Samples with missing data were removed from the analysis
		**(*d*) If applicable, explain how loss to follow-up was addressed**<br><br>Only subjects with complete series of data were included in the analysis
		**(*e*) Describe any sensitivity analyses**<br><br>Not applicable
**Results**		
Participants	13*	**(a) Report numbers of individuals at each stage of study—eg numbers potentially eligible, examined for eligibility, confirmed eligible, included in the study, completing follow-up, and analysed**<br><br>During the wellness study, 99 subjects completed the first year and 94 completed the second year. In the T2D study, 51 subjects completed the 3-month visit. All available plasma samples were analyzed using PEA-qPCR. For PEA-NGS analysis, we randomly selected 76 subjects with full longitudinal data and plasma samples at the start of the study (visit 1) and after approximately 15-18 months (visit 2) and 21-24 months (visit 3) in the wellness study, and 48 subjects with complete series of plasma samples in the T2D study.
		**(b) Give reasons for non-participation at each stage**<br><br>During the wellness study, 99 subjects completed the first year and 94 completed the second year. Since the trial spanned two years and each

		visit took almost a whole day of test, we are positively surprised about the outcome. In the T2D study, 51 subjects completed the 3-month visit.
		**(c) Consider use of a flow diagram**<br>Not needed.
Descriptive data	14*	**(a) Give characteristics of study participants (eg demographic, clinical, social) and information on exposures and potential confounders**<br><br>The complete list of assessed clinical variables is available in the supplementary materials.
		**(b) Indicate number of participants with missing data for each variable of interest**<br><br>Not applicable
		**(c) Summarise follow-up time (eg, average and total amount)**<br><br>The wellness study: the start of the study (visit 1) and after approximately 15-18 months (visit 2) and 21-24 months (visit 3)<br><br>The T2D study: the start of the trial (visit 1) and after approximately 1 month (visit 2) and 3 months (visit 3) of treatment.
Outcome data	15*	**Report numbers of outcome events or summary measures over time**<br><br>In total, 30 clinical chemistry together with anthropometric measurements were included in both wellness and T2D studies. In addition, insulin, C-peptide and the homeostatic model assessment of insulin resistance (HOMA-IR) were conducted in the T2D study.
Main results	16	**(*a*) Give unadjusted estimates and, if applicable, confounder-adjusted estimates and their precision (eg, 95% confidence interval). Make clear which confounders were adjusted for and why they were included**<br><br>Sex-related proteins: ANOVA analysis with age and visit as covariates.<br>pQTL analysis: linear regression model adjusted for age and sex at baseline.<br>differentially expression analysis: mixed-effect modelling with sex and age as covariates.<br><br>confounders with significant associations with protein expression or clinical outcomes were included in the statistical analysis
		**(*b*) Report category boundaries when continuous variables were categorized**<br><br>obesity group: BMI >30; non-obesity group: BMI <30<br>metformin responder group: FPG decrease >1; non-responder group: FPG decrease < 0.1; indeterminate group: FPG decrease between 0.1 and 1
		**(*c*) If relevant, consider translating estimates of relative risk into absolute risk for a meaningful time period**<br><br>Not applicable.
Other analyses	17	**Report other analyses done—eg analyses of subgroups and interactions, and sensitivity analyses**<br><br>Not applicable

Discussion

Key results	18	**Summarise key results with reference to study objectives**<br><br>We describe an approach suitable for comprehensive protein profiling, taking advantage from the observation that each individual has a unique protein profile and many proteins change during disease allowing stratification and monitoring of patients during treatment.
Limitations	19	**Discuss limitations of the study, taking into account sources of potential bias or imprecision. Discuss both direction and magnitude of any potential bias**<br><br>More in-depth analysis of the plasma protein profiles must be performed in larger disease cohorts to validate their use as clinical biomarkers
Interpretation	20	**Give a cautious overall interpretation of results considering objectives, limitations, multiplicity of analyses, results from similar studies, and other relevant evidence**<br><br>The wellness results presented here supports earlier observations that each individual has a unique blood protein "fingerprint" with larger inter-individual variations as compared to the intra-individual variation. Several novel proteins with strong association with known clinical parameters have here been identified and these are interesting to study in larger cohorts to validate them as clinical markers in routine settings. The genome association studies reported here also support earlier observations that many plasma protein levels in adult life are determined at birth by genetics and 69 novel genetic variants (pQTLs) not previously described were identified using the multiplex assay. This demonstrates that genetics should be taken into account when assessing an individual´s plasma protein levels in population studies.<br><br>The T2D results presented here support the notion that broad biochemical alterations are present already at the onset of type 2 diabetes and that protein profiling could deliver individualized health assessments of cardiometabolic diseases. The anthropometrical parameters of the T2D cohort allowed us to stratify the patients into two distinct groups based on BMI and an analysis of the protein profiles between the health individuals and the T2D patients at base line (visit 1) revealed several proteins that could be useful for early detection of disease based solely on protein profiles. The proteome profile allowed us to stratify the patients into responders and non-responders of naïve metformin drug treatment and analysis with a selected panel of proteins demonstrated that persons less likely to respond to treatment could be identified before the start of treatment. The suggestion that a panel of protein assays could be used to guide the physician regarding choice of drug treatment is highly promising, but it is important to point out that more in-depth analysis of these plasma protein profiles must be performed in larger disease cohorts to validate their use as clinical biomarkers.
Generalisability	21	**Discuss the generalisability (external validity) of the study results**<br><br>The results presented in the study using PEA-NGS supports the earlier observations in the relevant PEA-qPCR studies
**Other information**		
Funding	22	**Give the source of funding and the role of the funders for the present study and, if applicable, for the original study on which the present article is based**

	The main funding was provided from the Erling Persson Foundation (KCAP).

*Give information separately for exposed and unexposed groups.

Note: An Explanation and Elaboration article discusses each checklist item and gives methodological background and published examples of transparent reporting. The STROBE checklist is best used in conjunction with this article (freely available on the Web sites of PLoS Medicine at http://www.plosmedicine.org/, Annals of Internal Medicine at http://www.annals.org/, and Epidemiology at http://www.epidem.com/). Information on the STROBE Initiative is available at http://www.strobe-statement.org.

Supplementary Note 2. Clinical Study Protocol

The Wellness Profile – studies of the normal omics profile in healthy subjects

The Wellness Profile – studier av den normala omics-profilen hos friska individer

---

**Study Code:**	The Wellness Profile
**Version Number:**	1.0
**Date:**	2015-05-31
**Sponsor and Principal Investigator**	Göran Bergström

Table of Contents

1. Introduction 5
1.1. Background 5
1.2. Rationale for conducting The Wellness Profile Study 6
2. Study Objectives 7
3. Study population 7
3.1. Inclusion criteria 7
3.2. Exclusion criteria 7
3.3. Subject enrolment 7
3.4. Discontinuation and withdrawal of subjects 8
3.4.1. Premature termination of the study 8
4. Study Design and Procedures 8
4.1. Overall study design 8
4.2. Informed consent 9
4.3. Biological Sampling Procedures 9
4.3.1. Blood sampling 9
4.3.2. Urine sampling 9
4.3.3. Feacal sampling 9
4.3.4. Clinical Chemistry/hematology 9
4.3.5. Biobanking 9
4.3.6. CyTOF analysis 10
4.3.7. Affinity proteomics 10
4.3.8. Olink analysis 10
4.3.9. RNA sequencing 10
4.3.10. Whole genome sequencing 11
4.3.11. Metabolomics 11
4.3.12. Microbiomics 11
4.4. Anthropometry 11
4.4.1. Height 11
4.4.2. Weight 11
4.4.3. Waist circumference 12
4.4.4. Hip circumference 12
4.5. Bioimpedance 12
4.6. Blood pressure 12
4.7. Questionnaires 12
5. Statistics 13
5.1. Sample size calculation 13
6. Bioinformatics strategies 13
7. Data Management 13
7.1. Recording of data 13
7.1.1. Source data 14
7.2. Data storage and distribution 14
8. Quality Control and Quality Assurance 15
8.1. Monitoring 15
8.2. Qualifications 15

9. Study Follow-up using National Population Registries ... 15
10. Clinical follow-up ... 15
11. Ethics ... 16
11.1. Informed consent ... 16
11.2. Ethics committee ... 16
11.3. Insurances ... 16
11.4. Ethical considerations ... 16
12. Project organisation and infrastructure ... 17
12.1. Stakeholders and Executive joint committee ... 17
12.2. Report and publications ... 17
13. Study timetable ... 17
14. List of References ... 17
APPENDIX A: Transcriptome-wide allele specific expression in human primary white blood cells ... 19
APPENDIX B: to study how changes in transcription of mRNA in peripheral mononuclear cells (PBMC) correlate with changes in PBMC phenotype and systemic biomarkers of inflammation ... 22
APPENDIX C: Study the normal variation in the microbiome. ... 24

List of Abbreviations

Abbreviation	Explanation
ApoA1	Apolipoprotein A1
ApoB	Apolipoprotein B
ALAT	Alaninaminotransferas
ASE	Allele Specific Expression
BBMRI	Swedish Biobanking and Biomolecular Resources Research Infrastructure
COPD	Chronic Obstructive Pulmoranry Disease
CVD	Cardiovascular Disease
EC	Ethics Committee
EVF	Erythrocyte Volume Fraction
GC-MS	Gas Chromatography Masspectrometry
Hb	Hemoglobin
HbA1c	Glycated Hemoglobin
HDL	High Density Lipoprotein
hsCRP	High sensitivity C-reactive Protein
ICF	Informed Consent Form
LC-MS	Liquid Chromatography Masspectrometry
LDL	Low Density Lipoprotein
LIMS	Laboratory Information Management System
LPK	Leukocytplasmakoncentration
MCHC	Mean Corpuscular Hemoglobin Concentration
MCV	Mean Corpuscular Volume
MI	Myocardial Infarction
NCD	Non-communicable diseases
NT-proBNP	N-terminal of the prohormone brain natriuretic peptide (NT-proBNP)
p-Glucose	plasma Glucose
PBMC	Peripheral Blood Mononuclear Cells
PIN	Personal Identification Number
RBC	Red Blood Cell
SCAPIS	Swedish cardiopulmonary bioimage study
s-Cholesterol	serum Cholesterol
s-FE	Serum Ferritin
s-GT	Serum Glutamyl Transferase
s-TG	Serum Triglycerides
TIBC	Total Iron Binding Capacity
TNT	Troponin T
TPK	Thrombocyte particle concentration
WBC	White Blood Cells

1. INTRODUCTION

1.1. Background

Non-communicable diseases like cardiovascular disease (e.g. ischemic heart disease and stroke), cancer, chronic obstructive pulmonary disease (COPD), diabetes and dementia are the leading causes of morbidity and mortality in Sweden (Socialstyrelsen 2012) and globally. There is need for better understanding of the mechanism behind these diseases and improved diagnostic tools to enable more effective treatment and earlier detection.

Traditionally, in the diagnosis and treatment of these diseases a limited number of markers in the blood, urine or feces are measured. However, emerging new technologies have made it possible to measure a vast number of substances in the body with a simple blood, urine or fecal sample (Hood 2014). With massive parallel DNA sequencing the entire genome can be mapped in detail (genomics) and it is also possible to measure the expression of all genes into RNA (transcriptomics) in the white blood cells. A large number of the proteins encoded by these RNAs can be measured (proteomics) in the blood, while small molecules in the blood and urine can be measured by metabolomics or lipidomics. In addition, techniques for detailed mapping of the composition of the intestinal microbiota (metagenomics) have been developed. These technologies have seen a very rapid development in recent years and the cost of analyses have dropped dramatically. This trend is expected to continue in the future.

All of these "omics" technologies generate massive amounts of data. If multiple “omics” techniques are applied at the same time, the complexity will increase further, but so will also the opportunities to see patterns of changes in how these data vary. If a comprehensive omics analysis were to be made in connection with the development of disease or simultaneously with an intervention against disease, the coordinated pattern that it triggers will provide important information about the mechanisms behind the disease or effects of the intervention.

In 2012, we saw one of the first publications describing how such a comprehensive analysis was made (Chen Cell 2012). One (sic) single individual was followed for two years with repeated sampling from blood and urine. Extensive “omics” analysis of these samples could in detail describe coordinated patterns of changes in the “omics” profile associated with viral infections and changes in lifestyle. The result of Chen et al shows that omics profiling has great potential. If the technique is applied to subjects during disease development or during pharmacological interventions important mechanistic information can be derived. Other applications include identification of new biomarkers for early detection of disease, improved diagnostics, and personalized treatment.

In The Wellness Profile Study we want to examine the normal variation in an individual’s omics profile to determine the profile that is associated with health. In the current project, we define this as the spontaneous variation that occurs in the omics profile in clinically healthy individuals in the absence of changes in measurable environmental exposure. These spontaneous fluctuations can for example be explained by mechanisms such as genome wide allele specific expression (Rozowsky 2011) that are incompletely understood and will be specifically addressed in the current

study. To establish the normal Wellness Profile is an important first step since comprehensive molecular profiling at this level has not been done previously on a group level, only in single individuals (Chen et al Cell 2012). Once this wellness profile is established we can design and calculate study size for trials that will compare it to profiles associated with different disease states with the aim of developing new early markers of disease.

The current study is a sub-study of the Swedish CArdioPulmonary bioImage Study (SCAPIS), which is a nationwide, open-access, population-based cohort for the study of cardiovascular disease (CVD) and chronic obstructive pulmonary disease (COPD). SCAPIS will recruit and investigate 30,000 men and women aged 50 to 64 years with detailed imaging and functional analyses of the cardiovascular and pulmonary systems.

1.2. Rationale for conducting The Wellness Profile Study

While omics profiling appears to be a very promising tool, it is also evident that these profiles have a complex nature and are influenced by a number of factors. Also, the vast amount of data poses considerable challenges when it comes to data integration and visualization. Comprehensive omics profiling has not yet been tested in larger clinical studies, and at this stage there is a need for method development and validation.

Our main hypothesis for The Wellness Profile Study is that repeated, comprehensive and detailed omics profiling of an individual or group of individuals, can lead to the discovery of new mechanisms of disease and provide new markers for early detection of disease. We plan to test this hypothesis in several steps. The described study is a first step in which we want to lay the basis for future studies by developing a bioinformatics platform and studying the normal variation in the omics profile of healthy individuals. In later studies we will continue these efforts by examining the omics profile of individuals with specific diseases and/or exposed to specific interventions.

Results from this study will be presented descriptively, focusing on the natural variation of omics profiles over time. Any considerable change for an individual in for example health, diet, body composition or environmental exposure during the study will be assessed in relation to potential fluctuations in the omics profiles. These results will inform the design and sample size calculation of future studies.

To our knowledge, this is the largest amount of omics data generated in a study on humans. Therefore The Wellness Profile Study provides a unique opportunity to explore relationships within the data to propose new hypotheses on normal human biology. Below we have defined six objectives of this first study. More detailed descriptions of some of these objectives are given in Appendices A, B and C. Some of these objectives will be addressed in groups of individuals while others can be addressed at the level of each individual.

2. STUDY OBJECTIVES

1. To develop a bioinformatics platform for visualization and analyses of spatial and temporal changes in comprehensive data from omics profiling.
2. To describe the normal variation in the omics profile of healthy individuals (The Wellness Profile).
3. To study the natural variation of genome wide allele specific expression (See appendix A).
4. To study how changes in transcription of mRNA in peripheral blood mononuclear cells (PBMC) correlate with changes in PBMC phenotype and systemic biomarkers of inflammation (See Appendix B).
5. To explore how changes in health, diet, body composition or other environmental exposure correlate with changes in the omics profile at the individual level.
6. To study how changes in the gut microbiome correlates with changes in PBMC phenotype and systemic biomarkers of inflammation (See Appendix C).

3. STUDY POPULATION

3.1. Inclusion criteria

For inclusion in the study subjects must fulfil the following criteria

1. Signed informed consent to participate in the study.
2. Randomly selected and included in the Gothenburg SCAPIS cohort.
3. 50 but not yet 65 years of age at the time of selection from the SCAPIS cohort.
4. Ability to understand instructions and complete questionnaires, as judged by the study staff.

3.2. Exclusion criteria

1. Previously received health care for myocardial infarction, stroke, peripheral artery disease or diabetes.
2. Presence of any clinically significant disease which, in the opinion of the investigator, may interfere with the results or the subject´s ability to participate in the study.
3. Any major surgical procedure or trauma within 4 weeks of the first study visit.
4. Medication for hypertension or hyperlipidemia.

3.3. Subject enrolment

The Wellness Profile Study will follow 75-100 SCAPIS participants (included in the Gothenburg SCAPIS cohort) longitudinally. The subjects in SCAPIS are between 50-64 years and randomly selected from the Swedish population registry. This age group is selected since they will have several risk factors of disease but the level of prevalent disease will be low.

Eligibility is assessed at the first visit in SCAPIS in consecutive order. At the second visit in SCAPIS, eligible candidates receive information about The Wellness Profile Study and are asked for their interest in participating. A telephone call after 1 week confirms willingness to participate, and the candidate is scheduled for Visit 1 in the The Wellness Profile study within 8 weeks after the first SCAPIS study visit.

In order to achieve an equal gender distribution in the study, separate lists will be created with the interested subjects, one for women and one for men. The recruitment of subjects to the study will

be done by sequentially contacting subjects from the two lists, to continuously have an equal number of men and women scheduled for a first visit in the study.

3.4. Discontinuation and withdrawal of subjects

A subject can at any time withdraw from any study procedure or the entire study. This will not have any impact of the future care of the subject.

3.4.1. Premature termination of the study

The Executive joint committee may decide to stop the trial or part of the trial at any time. If the trial is prematurely terminated or suspended, the Ethics committee should be notified and provided a written explanation.

4. STUDY DESIGN AND PROCEDURES

4.1. Overall study design

A total of 75-100 subjects will be included in The Wellness Profile Study, aiming for at least 50 subjects with full data at all time-points at end of study. The drop-out rate will be continuously monitored and used to adjust the recruitment rate so that 50 subjects complete the study with full data collection. The examinations will be performed every third month during 9 months. Visit 1 should be performed within 8 weeks after visit 1 in SCAPIS. Whenever possible, all following visits should be scheduled every 3rd month (+/- 2 weeks). All subjects will be fasting overnight (at least 8 hours) before the visits. If a subject has not been fasting overnight, blood sampling should be rescheduled and performed preferably at an extra visit as close as possible to the scheduled visit.

As the first procedure of Visit 1, informed consent will be collected from the subject. When the informed consent has been signed, the subject will be registered in the study database. The other activities in the study are described in Table 1.

Table 1 Study Activities

	**Visit 1**	**Visit 2,3,4**
	Month 0	Month 3, 6, 9
Informed consent	X	
Clinical chemistry/hematology	X	X
Blood sample for Whole genome sequencing	X*	
Blood samples for affinity proteomics, Olink, metabolomics, CyTOF, RNA-seq	X	X
Urine sample for metabolomics	X	X
Faecal sample for microbiomics	X	X
Weight, waist, hip	X	X
Height	X	
Bioimpedance	X	X
Blood pressure	X	X
Questionnaires**	X	X

*Whole genome sequencing sample could be taken at a later visit
**In the SCAPIS study an extensive questionnaire was completed by all subjects. A selected sample of the questions from the main questionnaire will be completed in The Wellness Profile Study

4.2. Informed consent

At arrival for the first study visit, the subject is given full and adequate oral and written information about the nature, purpose, possible risks and benefits of the study. Subjects must also be notified that they are free to discontinue from the study at any time. The subject should be given the opportunity to ask questions and have sufficient time to consider the information provided. The subject's signed and dated informed consent must be obtained before conducting any procedure specifically for the study. The original informed consent form is stored at the study site and a copy is given to the subject.

4.3. Biological Sampling Procedures

4.3.1. Blood sampling

Samples for immediate laboratory analysis and for biobanking are obtained at the same time during all visits. Max 185 ml blood will be drawn from each subject in total during the study, max 50 ml at visit 1 and max 45 ml at each visit 2-4. Subjects should be fasting overnight (at least 8 hours).

4.3.2. Urine sampling

Spot-urine samples are collected at the study site.

4.3.3. Feacal sampling

Faeces will be sampled at home using dedicated devices and sent to the laboratory by ordinary mail. At arrival the sample will be frozen at minus 70°C for subsequent DNA extraction according to standard protocols.

4.3.4. Clinical Chemistry/hematology

Venous blood samples for immediate laboratory analysis of the following parameters are taken:
Capillary glucose (hemocue), p-Glucose, HbA1c, s-TG, s-Cholesterol, LDL, HDL, ApoB, ApoA1, ApoA1/B, Creatinine and hsCRP.
Hb, RBC, TPK, LPK, MCV, MCHC, EVF + WBC differential
ALAT, s-GT
Urate, Cystatin C, Vitamin D, TNT, NT-proBNP

4.3.5. Biobanking

Venous blood, spot-urine and faeces for biobanking are processed and stored in collaboration with Sahlgrenska Biobank using their standardized routines for handling and storage. As a quality marker, time from sampling to freezer is recorded i.e. time points for sampling and storage are noted/logged. All biobank samples are labelled with a code linked to the donor's personal identification number. In order to ensure complete traceability of samples and related information,

all codes and pre-analytical steps are controlled by the Biobank facility LIMS (Laboratory Information Management System).

4.3.6. CyTOF analysis

Sampling: 5 ml whole-blood (5 ml) is transferred into tubes prepared with Smart Tube protein stabilizer solution and immediately frozen at -80°C until analysis. Frozen tubes are transported to SciLifeLab, Stockholm.

Assays: Analysis using a 45-antibody panel to quantify all blood cell lineage frequencies and hundreds of cell subpopulations within these using novel bioinformatic tools (Brodin, 2014).

4.3.7. Affinity proteomics

Sampling: EDTA plasma is prepared from venous blood samples and one 225 µl aliqoute is frozen at -80°C within 3 hours post-blood draw. Samples are stored in Sahlgrenska Biobank at -80°C until transport. Frozen tubes are transported to SciLifeLab, Stockholm.

Assay/outcome: Proteomics will be untargeted detecting >10.000 protein fragments (Uhlen 2015). Direct labeling of samples and highly multiplexed and exploratory protein analysis using suspension bead arrays with multliple sets of 384 antibodies (Schwenk 2010). Proteomic analysis of auto-antibodies will be done with similar technique to find evidence of autoimmunity (Ayoglu, 2013).

4.3.8. Olink analysis

Sampling: EDTA plasma is prepared from venous blood samples and one 225 µl aliqoute is frozen at -80 C within 3 hours post-blood draw. Samples are stored in Sahlgrenska Biobank at -80°C until transport. Frozen tubes are transported to SciLifeLab, Stockholm.

Assay/outcome: Protein levels will be measured by the Proximity Extension Assay technique using the Proseek Multiplex CVD and Inflammation, 96x96 reagents kit (Olink Bioscience, Uppsala, Sweden) at the Clinical Biomarkers Facility, Science for Life Laboratory, Uppsala. Oligonucleotide-labeled antibody probe pairs are allowed to bind to their respective targets present in the plasma sample and addition of a DNA polymerase led to an extension and joining of the two oligonucleotides and formation of a PCR template. This technique will results in quantitative data on around 150 proteins related to cardiovascular disease and inflammation.

4.3.9. RNA sequencing

Sampling: PBMC are isolated from 5 ml heparinized whole blood within 2 hours of blood draw using FicollPaque/Lymphoprep. Alternative preparation is with Histopaque for separation of lymphocytes, monocytes and neutrophils to allow sequencing of specific subpopulations of PBMC. RNA preparation is performed at the Wallenberg laboratory in Gothenburg and samples are stored in the Sahlgrenska biobank at -80°C until transport. Frozen tubes are transported to SciLifeLab, Stockholm. RNA sequencing is performed at SciLifeLab.
Assay/outcome: Whole-transcriptome analysis with total RNA sequencing (total RNA-Seq) captures a broad range of gene expression changes and enables the detection of novel transcripts in both coding and non-coding RNA species.

4.3.10. Whole genome sequencing

Sampling: Whole blood samples (5 ml) are stored at -80°C in the Sahlgrenska biobank until transport to SciLifeLab. DNA preparation is performed at SciLife lab using Quiagen midiprepp.
Assay/outcome: Human Whole Genome Sequencing on Illumina X Ten system. We will use the standard setup for human whole genome sequencing which is paired-end sequencing (2x150bp) of one sample per lane with a coverage of at least 28X.

4.3.11. Metabolomics

Sampling: EDTA plasma is prepared from venous blood samples and one 100 μl aliqoute is transferred to a Saarstedt tube (no: 72.690.001, 1.5 mL) and frozen at -80°C within 3 hours post-blood draw. A 100 μl of the spot urine sample is aliquoted into a Saarstedt tube (no: 72.690.001, 1.5 mL) and immediately frozen at -80°C. Samples are stored in the Sahlgrenska biobank at -80°C until transport. Frozen tubes are transported to the Swedish Metabolomics Center, Umeå.
Assay/outcome: We will use a standard metabolomics analysis performed using both GC-MS and LC-MS (positive and negative electrospray ionisation). The analysis is based on untargeted MS-analysis and is not absolutely quantitative, i.e. the metabolite data are not expressed as nmol/ml plasma or nmol/mg tissue. Instead, the metabolite levels are expressed as normalised peak areas, which values can be compared between the analysed samples. Data will be delivered as peak areas of identified metabolites and of detected putative metabolites which are currently not identified.

4.3.12. Microbiomics

Sampling: Faecal sample (500 mg) are transferred into a sterilized tube (2 ml) and frozen at -80°C at arrival in Wallenberg laboratory, Gothenburg. DNA extraction is performed at Wallenberg laboratory, Gothenburg, following optimized protocols including beads (Sommer 2013).

Assay/outcome: Extracted DNA is sequenced (16S rRNA sequencing) in Gothenburg. Sequencing (16s RNA) and bioinformatics according to standard pipe-line developed in Gothenburg (Sommer 2013). In depth, shot-gun analyses will be decided on after analyses of initial results. If so, extracted DNA will be sent to SciLifeLab in Stockholm for sequencing at the Illumina Hiseq platform providing approximately 8 GB per sample (Sommer 2013). Dependent on which sequencing depth is used we can map most of the approximately 500–1,000 species that colonize the intestine (Sommer 2013)

4.4. Anthropometry

4.4.1. Height

The subject should be measured in indoor clothing to the nearest centimeter without shoes. Subjects should stand directly below the meter and keep their legs together, back straight and eyes straight ahead.

4.4.2. Weight

Weight should be measured on a calibrated balance beam or digital scale. Subjects should be dressed in light indoor clothing without shoes and be asked to empty pockets before weighing.

4.4.3. Waist circumference

All clothing except underwear should be removed to ensure correct positioning of the measuring tape. Subjects should stand erect with the abdomen relaxed, after exhalation, arms at the side, feet together, and weight equally divided over both legs. A non-stretchable tape should be placed at the waist midway between the palpated iliac crest and the palpated lowest rib margin in the left and right mid-axillary lines (at the natural waistline or narrowest part of the torso as seen anteriorly). The tape should be even, parallel to the floor, not twisted with the measurement scale facing outward. The assessor will be instructed to ensure that the tape is just touching the skin but not compressing the soft tissue.

4.4.4. Hip circumference

The subject should stand erect with arms at the sides, feet together and weight equally divided over both legs, only dressed in underwear. The measurement should be taken at the maximum circumference over the buttocks with a non-stretchable tape. The tape should be kept horizontal, even, not twisted with the measurement scale facing outward. The assessor will be instructed to ensure that the tape is just touching the skin but not compressing the soft tissue.

4.5. Bioimpedance

The body composition analyzer must not be used on subjects who have active implants (e.g. pacemakers, cochlear implants).

The subjects should take off their shoes and socks. Subjects should clean their feet from any dirt (e.g., fluff from socks) as it can work as a barrier, thereby increasing the resistance which affects the measurement. The subject should stand on the foot plates, grab handles and keep them a bit outside the body. The subject should stand still during the measurement. The arms should not touch the body and the upper thighs should not touch each other during the measurement.

4.6. Blood pressure

Brachial arterial blood pressure should be obtained by automatic measurement in both arms at visit 1 and thereafter in the arm that showed the highest blood pressure at visit 1 (Omron P10). Systolic and diastolic pressure is registered in supine position and after 5 minutes rest. Cuff size should be adjusted according to arm circumference. The cuff should be in level with the heart.

4.7. Questionnaires

A questionnaire, administered already in the SCAPIS trial, comprising 140 questions separated in sets relating to factors central to the research aims, has been designed to collect detailed information on self-reported health, family history, medication, occupational and environmental exposure, lifestyle, psychosocial well-being, socioeconomic status and other social determinants. A food-frequency questionnaire (Mini-Meal-Q) with 35 questions is also used in SCAPIS.
At each visit in the Wellness study a selection of questions are repeated that will update the information of the SCAPIS questionnaire. We will ask for changes in life-style factors between each

visit such as infections, disease, medication, exercise level etc. The food frequency questionnaire is repeated at all visits.
Questions about antibiotic intake before (6 months) and during the study will include treatment duration, type of antibiotics and if any probiotics have been taken.

5. STATISTICS

5.1. Sample size calculation

It is impossible to perform power calculations. Previous studies in this area has published data on one (sic!) individual (Chen Cell 2012). Studies on ASE has published data on up to 10 individuals (Edsgärd, in manuscript, 2015). We therefore anticipate that data from 50 subjects will be sufficient to characterize the Wellness profile and to meet our exploratory aims. Data will also be published based on individuals changes in omics profile in response to environmental changes (e.g. cold, medication, weight loss). All results from the study will only be presented descriptively.

For future studies, a power calculation based on knowledge collected in this study will be conducted to estimate the sample size.

6. BIOINFORMATICS STRATEGIES

The whole study will generate a wide variety of data types describing both the clinical and omics profile of healthy individuals over time. Novel bioinformatics approaches will be developed and explored to integrate data on clinical metadata with clinical chemistry data, proteomic profiles (including biomarkers for cardiovascular risk, inflammation and autoantibodies), transcript data from PBMCs, data on cell population frequency in blood, endogenous metabolite profiles, gut microbiome profiles and genetic data. This work will be done at SciLife Laboratories with a dedicated team of bioinformaticians.

We will analyze this wealth of Omics data using different approaches, such as previously described by Chen et al 2012. For each profile (transcriptome, proteome, metabolome, microbiome), we will systematically search for two types of nonrandom patterns: (1) correlated patterns over time and (2) single unusual events (i.e., spikes that may occur at any given time point defined as statistically significantly high or low signal instances compared to what would be expected by chance).

7. DATA MANAGEMENT

A selection of the results from the examinations will be presented to the participating subjects using a web portal developed by DaraLabs. This portal will also be used to enter or upload study data. The recording, storage and distribution of data are further explained in section 7.1.

7.1. Recording of data

The investigator will ensure that all data collected in the study, are recorded in a timely manner according to any instructions provided. Study data will be entered into a central database at the study site through an electronic system hosted by DaraLabs. The database will be hosted in a

secure professional hosting facility with audited appropriate physical and logical security levels for the stored data.

In case of database failure, the data may be recorded on a paper based source data document. Study data will be entered by study staff. The electronic questionnaires, to be completed by the subjects will be distributed through the portal. The questionnaire will be available for completion by the subject in advance before the study visits (except for visit 1) and the subjects are encouraged to complete the questionnaire before the visits. If it has not been completed at the time for the visit, the subject will have time to complete it on site. All system users, study personnel and study subjects, will have unique login details.

Results from the lab analysis will be uploaded to the database, and a selection of the results will be presented to subjects. The selection will be; length, weight, BMI, waist, hip, bio impedance (% fat), blood pressure, Hb, glucose, HbA1c, triglycerides, cholesterol, LDL, HDL.

The following safety measures will be taken to secure data quality:

- The electronic questionnaires, to be completed on an internet connected device (computer, tablet or smartphone) by study subjects, will be designed to make it as easy as possible for the subject to reply to all questions.
- At the completion of the study, the presence and distribution of all variables will be checked and compared intra-individually to detect any extreme outliers or obvious errors. Questionnaire data entered by the subjects themselves will not be analyzed/cleaned/excluded from the database through any similar procedure.

When these procedures are completed, a meeting with data management, a statistician and study management will be held. Questions on any data exclusions or corrections will be formally decided upon, clean file will be declared and the database locked. The reason for excluding any data declared as erroneous will be described in detail in the report from the meeting. The principal investigator is responsible for ensuring that a final clean file is declared and documented.

Data from The Wellness Profile trial will be merged with data from SCAPIS baseline examination.

7.1.1. Source data

Source data could be medical records, working sheets or be entered directly into the study database. The source will be defined in a source data document.

7.2. Data storage and distribution

The study organization at Sahlgrenska responsible for interactions with participants in the study will use electronic case report forms (eCRF) for study data documentation. No PIN number are used, subjects are identified by their study code.

Direct analyses of blood via Clinical Chemistry at Sahlgrenska University Hospital are done within the hospital and PIN number are therefore used. However, data is transferred to eCRF and thereafter only identified by study code. Biobanking is done via the Sahlgrenska Biobank according

standard routines within the hospital. Blood samples withdrawn from the biobank are labelled with study code only.

To directly interact with study participants a dedicated WEB-based application has been developed by DaraLabs AB. In this system, the study organization can communicate with participants and remind them of study visits and ask them to respond to questionnaires and give feed-back. The participants will also receive feed-back on selected study data via the application. These data are: length, weight, BMI, waist, hip, bioimpedance (% fat), blood pressure, Hb, glucose, HbA1c, triglycerides, cholesterol, LDL, HDL.

At the SciLifeLab, data will be stored at the KTH/School of Biotechnology in pass word protected systems physically held at the Albanova (KYH) (Stockholm).
Data will be analyzed and therefore also held at Department of Biology and Biological Engineering, Chalmers, Gothenburg (Prof. Jens Nielsens grupp).

Data generated within the study can be provided also to partners outside the project´s research team. Data can also be used in conjunction with partners in industry and companies. Data will be stored coded so identity cannot be revealed. The storage system will follow national guidelines for patient security and integrity for all data.

8. QUALITY CONTROL AND QUALITY ASSURANCE

8.1. Monitoring

All informed consents will be checked and the identity of the subject who signed the consent form will be verified.

8.2. Qualifications

All investigations will be performed by dedicated study staff. Study staff should be qualified to perform delegated tasks and be trained in study procedures.

9. STUDY FOLLOW-UP USING NATIONAL POPULATION REGISTRIES

No registry data will be used in this study.

10. CLINICAL FOLLOW-UP

The general recommendations for clinical follow-up are as follows:

- Immediate findings such as elevated plasma glucose levels and blood pressure elevation will be communicated to the subjects by the study nurse or study physician during a study visit and appropriate action according to hospital guidelines will be taken.
- A sample of the results from the lab analysis will be presented to the subjects in an individual web –site (see 7.1).
- Subjects who have isolated risk factors or deviating blood samples will be informed.
- Moderate elevations in risk factors will, as a rule, be managed by asking the subject to contact his or her primary care physician.

- Minor changes in risk factors will be communicated to the subjects on the web portal. Major changes will be communicated by of the study doctor / nurse using personal contact with the subjects using phone or letter.
- At the completion of the study each participant will receive a written summary of results from direct biochemical analyses and physical measurements.
- No action will be taken on results generated in the omics part of the study. This information is given in the Informed Consent Form (ICF).

11. ETHICS

11.1. Informed consent

The Principal Investigator at the centre will ensure that the subject is given full and adequate oral and written information about the nature, purpose and possible risks and benefits of the study. Subjects must also be notified that they are free to discontinue from the study at any time. The subject should be given the opportunity to ask questions and allowed time to consider the information provided.

The subject's signed and dated informed consent must be obtained before conducting any procedure specifically for the study.

The original, signed ICF must be stored in the Investigator's Study File. A copy of the signed ICF must be given to the subject.

If a protocol amendment requires a change to the ICF, the EC must approve modifications that lead to a revised ICF before the revised form is used.

11.2. Ethics committee

Before enrolment of any subject into The Wellness Profile trial, the final study protocol, including the final version of the Informed Consent Form a written approval must be approved or given a favorable opinion in writing by the Ethics Committee (EC) in Gothenburg. The principal investigator is responsible for informing the Ethics Committee of any modifications and amendments to the protocol as per local requirement. The EC must also be notified by the Sponsor or designee in writing of the interruption and/or completion of the study.

11.3. Insurances

Subjects participating in The Wellness Profile trial are managed within the health care system and thus covered by the Patient Insurance according to the Swedish Patient Injury Act.

11.4. Ethical considerations

There are no physical dangers involved in any of the procedures.

The integrity of the subjects needs to be secured. All data are used in coded format and in secure environments. We will in most cases publish data at group level to avoid identification of

individuals. However, we will most likely want to publish data also from individual cases. In this case vital data will be changed so that individuals cannot be recognized.

Participants will be informed on all data collected at study visits as this may help the participant to improve his/her risk profile.

Participants will not be informed on information on omics data since the clinical value is not known.

One ethical issue in this study is the theoretical possibility that a subject harbors a disease-causing mutation that may be revealed by whole genome sequencing. However the likelihood of any major disease-causing mutation is relatively low in a population of healthy subject over 50 years of age. Also, we will not apply bioinformatics strategies to actively search for clinically significant information, i.e. we will not filter the genetic data against databases of disease-causing genetic variants. Furthermore, a disease casing variant should only be communicated after it has been verified (i.e. Sanger sequencing in an accredited clinical genetics laboratory) which will not be done in this study. Therefore subject will be carefully informed (orally and in the ICF) that no genetic information will be returned in this study.

12. PROJECT ORGANISATION AND INFRASTRUCTURE

12.1. Stakeholders and Executive joint committee

The Wellness Profile trial is led by an executive joint committee comprising representatives from three stakeholders: SCAPIS (Sahlgrenska), SciLIfeLab (KTH Center for applied proteomics – KCAP) and BBMRI (Swedish Biobanking and Biomolecular Resources Research Infrastructure). Each stakeholder nominates one person (and one suppleant) to the executive committee. The executive joint committee has the overall scientific and fiscal responsibility for The WellnessProfile study.

12.2. Report and publications

Results will be published in per-reviewed scientific journals.

13. STUDY TIMETABLE

The Wellness Profile study is planned to start Q3 2015 in Gothenburg. The recruitment period is estimated to be 3 months and follow-up time 9 months. The study is expected to be completed by Q3 2016.

14. LIST OF REFERENCES

1 Socialstyrelsen. Causes of death 2011. Stockholm. 2012.
2 Chen R, Mias GI, Li-Pook-Than J, et al. Personal omics profiling reveals dynamic molecular and medical phenotypes. Cell 2012; 148: 1293-307.
3 Hood L, Price ND. Demystifying disease, democratizing health care. Science translational medicine 2014; 6: 225ed5.

4 Schwenk JM, Igel U, Neiman M, et al. Toward next generation plasma profiling via heat-induced epitope retrieval and array-based assays. Mol Cell Proteomics 2010; 9: 2497-507.
5 Ayoglu B, Haggmark A, Khademi M, Olsson T, Uhlen M, Schwenk JM, Nilsson P. Autoantibody profiling in multiple sclerosis using arrays of human protein fragments. Mol Cell Proteomics 2013; 12: 2657-72.
6 Rozowsky J, Abyzov A, Wang J, et al. AlleleSeq: analysis of allele-specific expression and binding in a network framework. Mol Syst Biol 2011; 7: 522.
7 Shekhar K, Brodin P, Davis MM, Chakraborty AK. Automatic Classification of Cellular Expression by Nonlinear Stochastic Embedding (ACCENSE). Proc Natl Acad Sci U S A 2014; 111: 202-7.
8 Sommer F, Backhed F. The gut microbiota - masters of host development and physiology. Nat Rev Microbiol 2013; 11: 227-38.

APPENDIX A: TRANSCRIPTOME-WIDE ALLELE SPECIFIC EXPRESSION IN HUMAN PRIMARY WHITE BLOOD CELLS

Background to the area
Recent advances in sequencing technologies have provided rich catalogs of genetic variation and a more detailed and accurate picture of gene expression to emerge. However, the functional role of genetic variation and its impact on expression variation remains largely unknown. Genome-wide association studies (GWAS) have shown that the majority of common genetic variants associated to complex diseases have a relatively modest effect and are mostly present in non-coding regions, indicating that these loci mediate their effect via cis-regulation of transcription [1]. To find cis-regulatory variants that affect transcription, expression quantitative trait locus (eQTL) analysis has been a common approach. Unfortunately, eQTL analyses are hampered by the need for many samples, inter-individual differences in expression, modest effects, as well as the large number of SNP-gene combinations [2].

Another approach to identify the presence of cis-regulatory variation is to study allele specific expression (ASE). ASE is the difference in expression between the paternal and maternal haplotype of a transcript within an individual. A significant advantage of ASE analysis compared to eQTL analysis is that it can be conducted on single individuals since the alternative alleles serve as within-sample controls, with identical cellular environment and trans-acting factors. A detected allelic imbalance in transcription could thus indicate a heterozygous variant within a binding site, causing differential binding of transcription factors or epigenetic modifiers [3-6], or at a splice site or UTR, affecting transcript processing [7]. Corroborating this, a study of 60 CEU HapMap individuals showed that genes exhibiting ASE are enriched for eQTLs [8], and the ENCODE project reported clear correlations between allele-specific epigenetic marks and allele-specific transcription [9]. Information about ASE could be used to reduce the number of genes in the genome for which regulatory regions should be investigated in functional or genetic association studies [10]. Thus, ASE analysis is a useful approach to further our understanding of the impact of genetic variation on cellular processes, and a natural step towards a more detailed map of transcription and transcriptional regulation.

RNA sequencing (RNA-seq) enables a comparably unbiased interrogation of the transcriptional landscape. It is suitable for detecting allele-specific expression, provided that the sequencing depth is sufficient for a statistical analysis with the desired power. Specifically, using RNA-seq data to assess ASE enables the detection of heterozygous variants within genes. Previous RNA-seq based studies of allele specific expression have reported that roughly 20% of heterozygous variants in coding regions of the human genome display ASE [11-13] and this is also the approximate prevalence in pre-RNA-seq ASE investigations [14-17] There is a span from 3-5% of SNPs with ASE [18, 19] to 45% of genes with ASE, where the highest estimates are from cell lines or cancer cells [20]. In fact, most ASE studies have been performed on cancer cell lines [12, 13, 19] [21] with only a few exceptions [22].

Little focus has been placed on the potentially differential ASE between individuals or between biological conditions within a single individual. Li et al. [20] investigated both a cell line and several cancer tissues, but they were primarily not interested in analyzing differences between samples or

conditions, and in the end pooled all their data within each category. Heap et al. [23] investigated ASE in human primary CD4+ T cells by RNA-seq of four individuals, and presented evidence of widespread ASE in individuals, along with estimated false discovery rates. They included both untreated and activated samples in their analysis, but they did not study the differences in ASE in cells from the same individual between the two conditions.

Identification of differential ASE in an individual was, however, part of the analysis of the personal omics profile of a single human subject published by Chen et al. [22]. The differential ASE was measured by contrasting the ASE at a single time point against the ASE at several other time points. This was an observational study and the subject was not in a controlled environment – thus a wide range of factors could have caused the differential ASE that was observed over time, and the study would be difficult to reproduce.

Recently we investigated condition-dependent, as well as condition-independent (static), ASE by deep transcriptome-wide sequencing (RNA-seq) of primary white blood cells from eight human individuals before and after the controlled induction of an inflammatory response by in vitro treatment with lipopolysaccharide (LPS) (Edsgard et al, submitted). The purpose was to identify genetic variation of importance in an inflammatory response. We developed methods for detection and analysis of condition-dependent ASE in single individuals, at both single variant and gene levels. Our results indicate ASE of 3.5% to 8.2% of all heterozygous variants per individual, however fully accurate determination would require to map it against individual whole genome sequence which we did not have. Furthermore, we do not know which of the induced or static ASE that varies naturally over time and to what extent, which makes it hard from the previous study to identify the more interesting ASE variants and candidate rSNPs against a natural time variation background in healthy subjects.

Project aim

As detailed above, there are a number of gaps in our understanding of the natural variation and the influence of different biological conditions on ASE. Therefore we propose to use the The Wellness Profile study to perform deep RNA seq of primary white blood cells at 4 consecutive time points 3 months apart in 50 individuals and to map this against the individual genomes.

The main objective of this project is to describe the inter-individual and time longitudinal variation in allele specific transcription in human primary white blood cells by combining consecutive deep RNA sequencing and whole genome sequencing.

References

1 Lander E. Initial impact of the sequencing of the human genome. Nature 2011; 470: 187-97.

2 Pastinen T. Genome-wide allele-specific analysis: insights into regulatory variation. Nature reviews Genetics 2010; 11: 533-8.

3 Rozowsky J, Abyzov A, Wang J, et al. AlleleSeq: analysis of allele-specific expression and binding in a network framework. Molecular systems biology 2011; 7.

4 Degner J, Pai A, Pique-Regi R, et al. DNase[thinsp]I sensitivity QTLs are a major determinant of human expression variation. Nature 2012; 482: 390-4.

5 Prendergast JG, Tong P, Hay D, Farrington S, Semple CA. A genome-wide screen in human embryonic stem cells reveals novel sites of allele-specific histone modification associated with known disease loci. Epigenetics & chromatin 2012; 5: 6.

6 Reddy T, Gertz J, Pauli F, et al. Effects of sequence variation on differential allelic transcription factor occupancy and gene expression. Genome research 2012; 22: 860-9.
7 Li G, Bahn J, Lee J-H, Peng G, Chen Z, Nelson S, Xiao X. Identification of allele-specific alternative mRNA processing via transcriptome sequencing. Nucleic Acids Research 2012.
8 Montgomery S, Sammeth M, Gutierrez-Arcelus M, et al. Transcriptome genetics using second generation sequencing in a Caucasian population. Nature 2010; 464: 773-7.
9 ENCODE Project Consortium. An integrated encyclopedia of DNA elements in the human genome. Nature 2012; 489: 57-74.
10 Wittkopp P, Haerum B, Clark A. Evolutionary changes in cis and trans gene regulation. Nature 2004; 430: 85-8.
11 Skelly DA, Johansson M, Madeoy J, Wakefield J, Akey JM. A powerful and flexible statistical framework for testing hypotheses of allele-specific gene expression from RNA-seq data. Genome Res 2011; 21: 1728-37.
12 Montgomery SB, Sammeth M, Gutierrez-Arcelus M, et al. Transcriptome genetics using second generation sequencing in a Caucasian population. Nature 2010; 464: 773-7.
13 Rozowsky J, Abyzov A, Wang J, et al. AlleleSeq: analysis of allele-specific expression and binding in a network framework. Mol Syst Biol 2011; 7: 522.
14 Serre D, Gurd S, Ge B, et al. Differential Allelic Expression in the Human Genome: A Robust Approach To Identify Genetic and Epigenetic Cis-Acting Mechanisms Regulating Gene Expression. PLoS Genet 2008; 4: e1000006.
15 Ge B, Pokholok D, Kwan T, et al. Global patterns of cis variation in human cells revealed by high-density allelic expression analysis. Nature genetics 2009; 41: 1216-22.
16 An integrated map of genetic variation from 1,092 human genomes. Nature 2012; 491: 56-65.
17 Zhang K, Li JB, Gao Y, et al. Digital RNA allelotyping reveals tissue-specific and allele-specific gene expression in human. Nature methods 2009; 6: 613-8.
18 Heap GA, Yang JH, Downes K, et al. Genome-wide analysis of allelic expression imbalance in human primary cells by high-throughput transcriptome resequencing. Hum Mol Genet 2010; 19: 122-34.
19 Degner JF, Marioni JC, Pai AA, Pickrell JK, Nkadori E, Gilad Y, Pritchard JK. Effect of read-mapping biases on detecting allele-specific expression from RNA-sequencing data. Bioinformatics 2009; 25: 3207-12.
20 Li G, Bahn JH, Lee JH, Peng G, Chen Z, Nelson SF, Xiao X. Identification of allele-specific alternative mRNA processing via transcriptome sequencing. Nucleic Acids Res 2012; 40: e104.
21 Vijaya Satya R, Zavaljevski N, Reifman J. A new strategy to reduce allelic bias in RNA-Seq readmapping. Nucleic Acids Research 2012.
22 Chen R, Mias G, Li-Pook-Than J, et al. Personal omics profiling reveals dynamic molecular and medical phenotypes. Cell 2012; 148: 1293-307.
23 Heap G, Yang J, Downes K, et al. Genome-wide analysis of allelic expression imbalance in human primary cells by high-throughput transcriptome resequencing. Human Molecular Genetics 2010; 19: 122-34.

APPENDIX B: TO STUDY HOW CHANGES IN TRANSCRIPTION OF MRNA IN PERIPHERAL MONONUCLEAR CELLS (PBMC) CORRELATE WITH CHANGES IN PBMC PHENOTYPE AND SYSTEMIC BIOMARKERS OF INFLAMMATION

We will perform an omics wellness profile specifically targeting the immunoprofile of healthy individuals over time. We will be focusing on the cellular immune system in peripheral blood and the inflammatory mediators and plasma proteins that both influence and reflect the composition of cell population.
Our analysis combines transcriptomic, proteomic, metabolomic and cellular immunophenotype data for the peripheral blood compartment.
We will examine detailed dynamic trends related directly to the physiological states of the individuals and changes in biological processes occurring during healthy and diseased states such as common benign infections (cold, flu etc) that is expected to occur in a proportion of the individuals during the monitoring period.

By employing immunophenotyping using mass flow cytometry (CytTOF) we can on an unprecedent scale delineate the different white blood cell populations based on expression of proteins and cell markers characteristic of different stages in hematopoiesis and lymphopoesis. By deep RNA sequencing, we can determine the integrated transcriptome profile of this cell population and the concurrent dynamics. By plasma proteomics (affinity proteomics and Olink analysis) we can analyze 1) secreted mediators and/or shedded protein antigens in plasma derived from these cells 2) the inflammatory mediators and markers in plasma derived from tissues and organs that influence these cells their compartments of origin (bonemarrow, lymphoid tissues, liver etc) in modulating the immunoprofile.

We will analyze this wealth of Omics data using different approaches, such as previously described by Chen et al 2012. For each profile (transcriptome, proteome, metabolome), we will also systematically search for two types of nonrandom patterns: (1) correlated patterns over time and (2) single unusual events (i.e., spikes that may occur at any given time point defined as statistically significantly high or low signal instances compared to what would be expected by chance).

In the The Wellness Profile study we aim to understand the normal variation of molecular profiles in healthy individuals over time with the goal to facilitate a molecular definition of health and wellness (Hood 2015). The goal is to reveal biological networks that specify health and wellness so that we in later studies can see how these patterns are altered in aging and disease. The study described here will be used to develop and explore the bioinformatics approaches for systems biology data integration. Temporal molecular profiles will be used to study the normal variation within individuals over time and the variation of molecular pattern between the individuals.

References

Chen R, Mias G, Li-Pook-Than J, Jiang L, Lam H, Chen R, Miriami E, Karczewski K, Hariharan M, Dewey F et al. 2012. Personal Omics Profiling Reveals Dynamic Molecular and Medical Phenotypes. Cell 148(6): 1293-1307.

Hood L, Lovejoy JC, Price ND. BMC Med. 2015 Jan 9;13:4. doi: 10.1186/s12916-014-0238-7. Integrating big data and actionable health coaching to optimize wellness.

APPENDIX C: STUDY THE NORMAL VARIATION IN THE MICROBIOME.

While the sequencing of the human genome has provided invaluable knowledge, it is very difficult to change our own genetic makeup. The human microbiome, in contrast, is much more easily changed through simple means such as healthful probiotic cultures, bacteriotherapy, diet and other lifestyle interventions [1]. We are just beginning to learn the effects our microbiome has on us, but it is clear that they can be profound. Different properties of the microbiome have been linked to weight gain, inflammatory bowel disease, colon cancer and other gut disorders, rheumatoid arthritis, hypertension, eczema and autism [1]. Infant health even appears to benefit from a proper seeding of microbes at birth, with health consequences ranging into adolescence [2].
Consequently, the human microbiome may be as important to our health as the human genome. In the The Wellness Profile Study we aim to explore the gut microbiome in healthy individuals over time, since there is a need to clarify what is normal in the human microbiome in order to define a dysbiotic profile and thereby provide therapy alternatives or lifestyle interventions to change it back to normal again.
The next generation sequencing effort will focus on quantification of known microbial diversity and how the ratios of taxonomical groups correlate with external factors such as inflammation. DNA sequencing will thus concentrate on marker genes for taxonomy (i.e. 16S rRNA) and clinically interesting genes such as virulence coding genes (toxins, adhesins etc) and resistance factors (the “resistome”). After taxonomical analyses based on 16S rRNA sequencing, a selected number of samples will be metagenomically sequenced to chart important organisms that are distantly related to organisms in public databases. Faecal samples are superior to biopsy samples for metagenomic sequencing since they contain less human DNA.
A future step will be to include a set of demonstration projects to determine the relationship between human health and changes in the human gut microbiome (based on a reliable healthy gut microbiome data set). We will then get an opportunity to examine the relationship between changes in the human gut microbiome and diseases of interest.

References

1 Sommer F, Backhed F. The gut microbiota - masters of host development and physiology. Nat Rev Microbiol 2013; 11: 227-38.
2 Hesla HM, Stenius F, Jaderlund L, Nelson R, Engstrand L, Alm J, Dicksved J. Impact of lifestyle on the gut microbiota of healthy infants and their mothers-the ALADDIN birth cohort. FEMS Microbiol Ecol 2014; 90: 791-801.

Supplementary Note 3. Clinical Study Protocol

The T2D Profile - Studies of the omics profile in Type 2 Diabetes

(Studier av den molekylära profilen vid Typ 2 Diabetes)

**Study Code:**	The T2D Profile
**Version Number:**	1.0
**Date:**	2016-05-09
**Sponsor and Principal Investigator**	Göran Bergström

Table of Contents

1. Introduction ... 5
1.1. Background ... 5
1.2. Rationale for conducting The Molecular Profile of T2D Study ... 5
2. Study Objectives ... 6
3. Study population ... 7
3.1. Inclusion criteria ... 7
3.2. Exclusion criteria ... 7
3.3. Subject enrolment ... 7
3.4. Discontinuation and withdrawal of subjects ... 7
3.4.1. Premature termination of the study ... 7
4. Study Design and Procedures ... 7
4.1. Overall study design ... 7
4.2. Informed consent ... 8
4.3. Biological Sampling Procedures ... 9
4.3.1. Blood sampling ... 9
4.3.2. Urine sampling ... 9
4.3.3. Feacal sampling ... 9
4.3.4. Clinical Chemistry/hematology ... 9
4.3.5. Biobanking ... 9
4.3.6. CyTOF analysis ... 9
4.3.7. Affinity proteomics ... 10
4.3.8. Olink analysis ... 10
4.3.9. RNA sequencing ... 10
4.3.10. Whole genome sequencing ... 10
4.3.11. Metabolomics ... 11
4.3.12. Microbiomics ... 11
4.4. Anthropometry ... 11
4.4.1. Height ... 11
4.4.2. Weight ... 11
4.4.3. Waist circumference ... 11
4.4.4. Hip circumference ... 12
4.5. Bioimpedance ... 12
4.6. Blood pressure ... 12
4.7. Questionnaires ... 12
5. Statistics ... 13
5.1. Sample size calculation ... 13
6. Bioinformatics strategies ... 13
7. Data Management ... 13
7.1. Recording of data ... 13
7.1.1. Source data ... 14
7.2. Data storage and distribution ... 14
8. Quality Control and Quality Assurance ... 15
8.1. Monitoring ... 15
8.2. Qualifications ... 15

9. Study Follow-up using National Population Registries 15
10. Clinical follow-up 15
11. Ethics 16
11.1. Informed consent 16
11.2. Ethics committee 16
11.3. Insurances 16
11.4. Ethical considerations 16
12. Project organisation and infrastructure 17
12.1. Stakeholders and Executive joint committee 17
12.2. Report and publications 17
13. Study timetable 17
14. List of References 17

List of Abbreviations

Abbreviation	Explanation
ApoA1	Apolipoprotein A1
ApoB	Apolipoprotein B
ALAT	Alaninaminotransferas
BBMRI	Swedish Biobanking and Biomolecular Resources Research Infrastructure
COPD	Chronic Obstructive Pulmoranry Disease
CVD	Cardiovascular Disease
EC	Ethics Committee
EVF	Erythrocyte Volume Fraction
Hb	Hemoglobin
HbA1c	Glycated Hemoglobin
HDL	High Density Lipoprotein
hsCRP	High sensitivity C-reactive Protein
LDL	Low Density Lipoprotein
LIMS	Laboratory Information Management System
LPK	Leukocytplasmakoncentration
MCHC	Mean Corpuscular Hemoglobin Concentration
MCV	Mean Corpuscular Volume
MI	Myocardial Infarction
NCD	Non-communicable diseases
p-Glucose	plasma Glucose
PBM	Peripheral Blood Mononuclear Cells
PIN	Personal Identification Number
RBC	Red Blood Cell
SCAPIS	Swedish cardiopulmonary bioimage study
s-Cholesterol	serum Cholesterol
s-FE	Serum Ferritin
s-GT	Serum Glutamyl Transferase
s-TG	Serum Triglycerides
T2D	Type 2 diabetes
TIBC	Total Iron Binding Capacity
TPK	Thrombocyte particle concentration
TPK	Thrombocyte particle concentration

1. INTRODUCTION

1.1. Background

Non-communicable diseases like cardiovascular disease (e.g. ischemic heart disease and stroke), cancer, chronic obstructive pulmonary disease (COPD), diabetes and dementia are the leading causes of morbidity and mortality in Sweden (Socialstyrelsen 2012) and globally. There is need for better understanding of the mechanism behind these diseases and improved diagnostic tools to enable more effective treatment and earlier detection.

Traditionally, in the diagnosis and treatment of these diseases a limited number of markers in the blood, urine or feces are measured. However, emerging new technologies have made it possible to measure a vast number of substances in the body with a simple blood, urine or fecal sample (Hood et al, 2014). With massive parallel DNA sequencing the entire genome can be mapped in detail (genomics) and it is also possible to measure the expression of all genes into RNA (transcriptomics) in the white blood cells. A large number of the proteins encoded by these RNAs can be measured (proteomics) in the blood, while small molecules in the blood and urine can be measured by metabolomics or lipidomics. In addition, techniques for detailed mapping of the composition of the intestinal microbiota (metagenomics) have been developed. These technologies have seen a very rapid development in recent years and the cost of analyses have dropped dramatically. This trend is expected to continue in the future.

All of these "omics" technologies generate massive amounts of data. If multiple “omics” techniques are applied at the same time, the complexity will increase further, but so will also the opportunities to see patterns of changes in how these data vary. If a comprehensive omics analysis were to be made in connection with the development of disease or simultaneously with an intervention against disease, the coordinated pattern that it triggers will provide important information about the mechanisms behind the disease or effects of the intervention.

In 2012, we saw one of the first publications describing how such a comprehensive analysis was made (Chen et al, 2012). One (sic) single individual was followed for two years with repeated sampling from blood and urine. Extensive “omics” analysis of these samples could in detail describe coordinated patterns of changes in the “omics” profile associated with viral infections and changes in lifestyle. The result of Chen et al shows that omics profiling has great potential. If the technique is applied to subjects during disease development or during pharmacological interventions important mechanistic information can be derived. Other applications include identification of new biomarkers for early detection of disease, improved diagnostics, and personalized treatment.

1.2. Rationale for conducting The Molecular Profile of T2D Study

Our main hypothesis for The Molecular Profile of T2D Study is that repeated, comprehensive and detailed omics profiling of an individual or group of individuals, can lead to the discovery of new mechanisms of disease and provide new markers for early detection of disease. We plan to test

this hypothesis in several steps. The first step was to study the normal variation in the omics profile of healthy individuals, and this step has already been taken in the ongoing Wellness Profile Study (DNR 407-15) that started in October 2015 and will be finished in October 2016. In the Wellness Profile study we are assessing up to 100 healthy subjects every third month for 9 months with extensive omics-profiling, with the main aim to study the normal variation in the omics profile of healthy individuals. Work is currently ongoing to develop the bioinformatics platform that will integrate the large amount of data that is being generated.

The T2D Profile study described in this protocol is the second step in our omics profiling strategy. Here we aim to show proof-of-principle for omics-profiling of specific diseases, i.e. that integrated omics-profiling can be used to study a specific disease state and to study the effects of treatment. We have chosen to study T2D for two main reasons: 1) T2D is a systemic disease with glycemic stress that is strongly associated with both micro- and macrovascular complications in several organs. Thus there is a high likelihood that this is a disease state that will have a strong impact on the omics profile. 2) The metabolic derangements of T2D can be effectively treated with lifestyle- and pharmacological interventions which gives us an excellent opportunity to study changes in the omics profile when these subjects are metabolically normalized towards the more healthy state. From this wealth of omics data we hope to formulate new hypotheses around pathophysiological mechanisms of T2D and its complications.

The design of the T2D Profile study will very similar to the previous Wellness Profile study, except that this time we will include subjects with newly diagnosed T2D who will receive diabetes treatment during the course of the study. Results from this study will be presented descriptively, focusing on the difference between T2D patients and the healthy controls (obtained from the Wellness profile study), and the difference in omics-profiles before and after T2D treatment. In addition we also plan to combine data from this study with data from the previous Wellness Profile study to strengthen the statistical power when exploring relationships within the data. These explorations are defined in section 2 below and described in more detail in the protocol of the initial Wellness Profile study.

2. STUDY OBJECTIVES

1. To characterize the omics profile in T2D-patients versus healthy controls.
2. To study changes in the omics profile in T2D-patients when their metabolic status improves.

Data from the T2D Profile study will also be used to strengthen the analysis of objectives previously defined in the Wellness study:

3. To study the natural variation of genome wide allele specific expression.
4. To study how changes in transcription of mRNA in peripheral blood mononuclear cells (PBMC) correlate with changes in PBMC phenotype and systemic biomarkers of inflammation.
5. To explore how changes in health, diet, body composition or other environmental exposure correlate with changes in the omics profile at the individual level.

6. To study how changes in the gut microbiome correlates with changes in PBMC phenotype and systemic biomarkers of inflammation.

3. STUDY POPULATION

3.1. Inclusion criteria

For inclusion in the study subjects must fulfil the following criteria

1. Signed informed consent to participate in the study.
2. Age 50-65 years.
3. T2D diagnosis (the combination of either fasting p-glucose ≥7.0 mmol/L or an oral glucose tolerance test 2 hour p-glucose ≥11.1 mmol/L [or ≥12.2 mmol/L if measured capillary] at two separate occasions).

3.2. Exclusion criteria

1. Diabetes medication before study start.
2. Severe hyperglycemia requiring hospitalization or immediate insulin treatment, as judged by the investigator.
3. Presence of any clinically significant disease which, in the opinion of the investigator, may interfere with the subject´s ability to participate in the study.
4. Any major surgical procedure or trauma within 4 weeks of the first study visit.

3.3. Subject enrolment

Participants will be recruited from two ongoing studies: the SCAPIS (Swedish CArdioPulmonary bioImage Study, DNR 2010-228-31M, Umeå) and the IGT-Microbiota trial (Impaired glucose tolerance and gut microbiota DNR 560:13, Göteborg). Subjects who are diagnosed with T2D in any of these studies will be asked about their interest to participate in The T2D Profile Study and eligible subjects will be scheduled for Visit 1.

3.4. Discontinuation and withdrawal of subjects

A subject can at any time withdraw from any study procedure or the entire study. This will not have any impact of the future care of the subject.

3.4.1. Premature termination of the study

The Executive joint committee may decide to stop the trial or part of the trial at any time. If the trial is prematurely terminated or suspended, the Ethics committee should be notified and provided a written explanation.

4. STUDY DESIGN AND PROCEDURES

4.1. Overall study design

Up to 50 subjects will be included in The Molecular Profile of T2D Study, aiming for at least 25 subjects with full data at all time-points at end of study. The drop-out rate will be continuously monitored and used to adjust the recruitment rate so that at least 25 subjects complete the study with full data collection.

Study duration is 3 months in total. Study activities are described in Table 1 below. As the first procedure of Visit 1, informed consent will be collected from the subject. When the informed consent has been signed, the subject will be registered in the study database. Samplings for omics-profiling and related activities will be conducted at baseline and month 1 and 3.

During the study, all participants will be offered T2D management by a specialist in diabetology at the Sahlgrenska University Hospital according to current guidelines for T2D management (Socialstyrelsen 2015, Läkemedelsverket 2014). Treatment will be individualized based on each subject´s medical needs and is not specified by this protocol, and the treatment goals will be the same as in regular healthcare, i.e. to reduce HbA1c to <52 mmol/mol and to reduce other cardiovascular risk factors such as hypertension, obesity, dyslipidemia and smoking. After study completion, subjects will be referred to primary care for further T2D management. The baseline visit should be conducted before start of diabetes treatment.

All subjects will be fasting overnight (at least 8 hours) before the visits. If a subject has not been fasting overnight, blood sampling should be rescheduled and performed preferably at an extra visit as close as possible to the scheduled visit.

Table 1 Study Activities

	**Visit 1**	**Visit 2**	**Visit 3**
	Baseline	Month 1	Month 3
Informed consent	X		
Clinical chemistry/hematology	X	X	X
Blood sample for Whole genome sequencing	X*		
Blood samples for affinity proteomics, Olink, metabolomics, CyTOF, RNA-seq	X	X	X
Urine sample for metabolomics	X	X	X
Faecal sample for microbiomics	X	X	X
Weight, waist, hip	X	X	X
Height	X		
Bioimpedance	X	X	X
Blood pressure	X	X	X
Questionnaires**	X	X	X

*Whole genome sequencing sample could be taken at a later visit

**In the SCAPIS and IGT Microbiota trial an extensive questionnaire was completed by all subjects. A selected sample of the questions from the main questionnaire will be completed in The Molecular Profile of T2D Study

4.2. Informed consent

At arrival for the first study visit, the subject is given full and adequate oral and written information about the nature, purpose, possible risks and benefits of the study. Subjects must also be notified

that they are free to discontinue from the study at any time. The subject should be given the opportunity to ask questions and have sufficient time to consider the information provided. The subject's signed and dated informed consent must be obtained before conducting any procedure specifically for the study. The original informed consent form is stored at the study site and a copy is given to the subject.

4.3. Biological Sampling Procedures

4.3.1. Blood sampling

Samples for immediate laboratory analysis and for biobanking are obtained at the same time during all visits. Max 140 ml blood will be drawn from each subject in total during the study. Subjects should be fasting overnight (at least 8 hours).

4.3.2. Urine sampling

Spot-urine samples are collected at the study site.

4.3.3. Feacal sampling

Faeces will be sampled at home using dedicated devices and sent to the laboratory by ordinary mail. At arrival the sample will be frozen at minus 70 for subsequent DNA extraction according to standard protocols.

4.3.4. Clinical Chemistry/hematology

Venous blood samples for immediate laboratory analysis of the following parameters are taken: Capillary glucose (hemocue), p-Glucose, HbA1c, s-TG, s-Cholesterol, LDL, HDL, ApoB, ApoA1, ApoA1/B, hsCRP, Na, K, Creatinine, Hb, RBC, TPK, LPK, MCV, MCHC, EVF, WBC differential, ALAT, ASAT, ALP, Bil, s-GT, Urate, Cystatin C, Vitamin D, TNT, NT-proBNP and U-Alb/Creatinine.

4.3.5. Biobanking

Venous blood, spot-urine and faeces for biobanking are processed and stored in collaboration with Sahlgrenska Biobank using their standardized routines for handling and storage. As a quality marker, time from sampling to freezer is recorded i.e. time points for sampling and storage are noted/logged. All biobank samples are labelled with a code linked to the donor's personal identification number. In order to ensure complete traceability of samples and related information, all codes and pre-analytical steps are controlled by the Biobank facility LIMS (Laboratory Information Management System).

4.3.6. CyTOF analysis

Sampling: 5 ml whole-blood (5 ml) is transferred into tubes prepared with Smart Tube protein stabilizer solution and immediately frozen at -80°C until analysis. Frozen tubes are transported to SciLifeLab, Stockholm.

Assays: Analysis using a 45-antibody panel to quantify all blood cell lineage frequencies and hundreds of cell subpopulations within these using novel bioinformatic tools (Brodin et al, 2014).

4.3.7. Affinity proteomics

Sampling: EDTA plasma is prepared from venous blood samples and one 225 µl aliqoute is frozen at -80°C within 3 hours post-blood draw. Samples are stored in Sahlgrenska Biobank at -80°C until transport. Frozen tubes are transported to SciLifeLab, Stockholm.

Assay/outcome: Proteomics will be untargeted detecting >10.000 protein fragments (Uhlén 2015). Direct labeling of samples and highly multiplexed and exploratory protein analysis using suspension bead arrays with multliple sets of 384 antibodies (Schwenk 2010). Proteomic analysis of auto-antibodies will be done with similar technique to find evidence of autoimmunity (Ayoglu 2013).

4.3.8. Olink analysis

Sampling: EDTA plasma is prepared from venous blood samples and one 225 µl aliqoute is frozen at -80 C within 3 hours post-blood draw. Samples are stored in Sahlgrenska Biobank at -80°C until transport. Frozen tubes are transported to SciLifeLab, Stockholm.

Assay/outcome: Protein levels will be measured by the Proximity Extension Assay technique using the Proseek Multiplex CVD and Inflammation, 96x96 reagents kit (Olink Bioscience, Uppsala, Sweden) at the Clinical Biomarkers Facility, Science for Life Laboratory, Uppsala. Oligonucleotide-labeled antibody probe pairs are allowed to bind to their respective targets present in the plasma sample and addition of a DNA polymerase led to an extension and joining of the two oligonucleotides and formation of a PCR template. This technique will results in quantitative data on around 150 proteins related to cardiovascular disease and inflammation.

4.3.9. RNA sequencing

Sampling: PBMC are isolated from 5 ml heparinized whole blood within 2 hours of blood draw using FicollPaque/Lymphoprep. Alternative preparation is with Histopaque for separation of lymphocytes, monocytes and neutrophils to allow sequencing of specific subpopulations of PBMC. RNA preparation is performed at the Wallenberg laboratory in Gothenburg and samples are stored in the Sahlgrenska biobank at -80°C until transport. Frozen tubes are transported to SciLifeLab, Stockholm. RNA sequencing is performed at SciLifeLab.

Assay/outcome: Whole-transcriptome analysis with total RNA sequencing (total RNA-Seq) captures a broad range of gene expression changes and enables the detection of novel transcripts in both coding and non-coding RNA species.

4.3.10. Whole genome sequencing

Sampling: Whole blood samples (5 ml from each visit) are stored at -80°C in the Sahlgrenska biobank until transport to SciLifeLab. DNA preparation is performed at SciLife lab using Quiagen midiprepp.

Assay/outcome: Human Whole Genome Sequencing on Illumina X Ten system. We will use the standard setup for human whole genome sequencing which is is paired-end sequencing (2x150bp) of one sample per lane with a coverage of at least 28X.

4.3.11. Metabolomics

Sampling: EDTA plasma is prepared from venous blood samples and one 100 μl aliqoute is transferred to a Saarstedt tube (no: 72.690.001, 1.5 mL) and frozen at -80°C within 3 hours post-blood draw. A 100 μl of the spot urine sample is aliquoted into a Saarstedt tube (no: 72.690.001, 1.5 mL) and immediately frozen at -80°C. Samples are stored in the Sahlgrenska biobank at -80°C until transport. Frozen tubes are transported to the Swedish Metabolomics Center, Umeå.

Assay/outcome: We will use a standard metabolomics analysis performed using both GC-MS and LC-MS (positive and negative electrospray ionisation). The analysis is based on untargeted MS-analysis and is not absolutely quantitative, i.e. the metabolite data are not expressed as nmol/ml plasma or nmol/mg tissue. Instead, the metabolite levels are expressed as normalised peak areas, which values can be compared between the analysed samples. Data will be delivered as peak areas of identified metabolites and of detected putative metabolites which are currently not identified.

4.3.12. Microbiomics

Sampling: Faecal sample (500 mg) are transferred into a sterilized tube (2 ml) and frozen at -80°C at arrival in Wallenberg laboratory, Gothenburg. DNA extraction is performed at Wallenberg laboratory, Gothenburg, following optimized protocols including beads (Sommer 2013).

Assay/outcome: Extracted DNA is sequenced (16S rRNA sequencing) in Gothenburg. Sequencing (16s RNA) and bioinformatics according to standard pipe-line developed in Gothenburg (ref). In depth, shot-gun analyses will be decided on after analyses of initial results. If so, extracted DNA will be sent to SciLifeLab in Stockholm for sequencing at the Illumina Hiseq platform providing approximately 8 GB per sample (Sommer 2013). Dependent on which sequencing depth is used we can map most of the approximately 500–1000 species that colonize the intestine (Sommer 2013)

4.4. Anthropometry

4.4.1. Height

The subject should be measured in indoor clothing to the nearest centimeter without shoes. Subjects should stand directly below the meter and keep their legs together, back straight and eyes straight ahead.

4.4.2. Weight

Weight should be measured on a calibrated balance beam or digital scale. Subjects should be dressed in light indoor clothing without shoes and be asked to empty pockets before weighing.

4.4.3. Waist circumference

All clothing except underwear should be removed to ensure correct positioning of the measuring tape. Subjects should stand erect with the abdomen relaxed, after exhalation, arms at the side, feet together, and weight equally divided over both legs. A non-stretchable tape should be placed at the waist midway between the palpated iliac crest and the palpated lowest rib margin in the left and right mid-axillary lines (at the natural waistline or narrowest part of the torso as seen

anteriorly). The tape should be even, parallel to the floor, not twisted with the measurement scale facing outward. The assessor will be instructed to ensure that the tape is just touching the skin but not compressing the soft tissue.

4.4.4. Hip circumference

The subject should stand erect with arms at the sides, feet together and weight equally divided over both legs, only dressed in underwear. The measurement should be taken at the maximum circumference over the buttocks with a non-stretchable tape. The tape should be kept horizontal, even, not twisted with the measurement scale facing outward. The assessor will be instructed to ensure that the tape is just touching the skin but not compressing the soft tissue.

4.5. Bioimpedance

The body composition analyzer must not be used on subjects who have active implants (e.g. pacemakers, cochlear implants).The subjects should take off their shoes and socks. Subjects should clean their feet from any dirt (e.g., fluff from socks) as it can work as a barrier, thereby increasing the resistance which affects the measurement. The subject should stand on the foot plates, grab handles and keep them a bit outside the body. The subject should stand still during the measurement. The arms should not touch the body and the upper thighs should not touch each other during the measurement.

4.6. Blood pressure

Brachial arterial blood pressure should be obtained by automatic measurement in both arms at visit 1 and thereafter in the arm that showed the highest blood pressure at visit 1 (Omron P10). Systolic and diastolic pressure is registered in supine position and after 5 minutes rest. Cuff size should be adjusted according to arm circumference. The cuff should be in level with the heart.

4.7. Questionnaires

A questionnaire, administered already in the SCAPIS or in the IGT Microbiota trial, comprising 140 questions separated in sets relating to factors central to the research aims, has been designed to collect detailed information on self-reported health, family history, medication, occupational and environmental exposure, lifestyle, psychosocial well-being, socioeconomic status and other social determinants. A food-frequency questionnaire (Mini-Meal-Q) with 35 questions is also used in SCAPIS and the IGT Microbiota trial [35].

At all visits in the T2D Profile study, a selection of questions are repeated that will update the information of the original questionnaire. We will ask for changes in life-style factors between each visit such as infections, disease, medication, exercise level etc. The food frequency questionnaire is repeated at all visits. Questions about antibiotic intake before (6 months) and during the study will include treatment duration, type of antibiotics and if any probiotics have been taken. Questionnaires can be found in appendix 5 of ethics application.

5. STATISTICS

5.1. Sample size calculation

At this early stage of omics profiling it is impossible to perform power calculations. Previous studies in this area have published data on one (sic!) individual (Chen et al, 2012). Studies on ASE have published data on up to 10 individuals (Edsgärd et al, 2016). We therefore anticipate that complete data from 15-30 T2D subjects, in combination with data from the 50-100 non-diabetic controls in the previous Wellness Profile Study, will be sufficient to characterize the omics profile of T2D. All results from the study will only be presented descriptively.

6. BIOINFORMATICS STRATEGIES

The whole study will generate a wide variety of data types describing both the clinical and omics profile over time. Novel bioinformatics approaches will be developed and explored to integrate data on clinical metadata with clinical chemistry data, proteomic profiles (including biomarkers for cardiovascular risk, inflammation and autoantibodies), transcript data from PBMCs, data on cell population frequency in blood, endogenous metabolite profiles, gut microbiome profiles and genetic data. This work will be done at SciLife Laboratories with a dedicated team of bioinformaticians.

Results from this study will be presented descriptively, focusing on the difference between T2D patients and the healthy controls obtained from the first Wellness profile study, and the difference in omics-profiles before and after T2D treatment. In addition we also plan to combine data from this study with data from the previous Wellness Profile study to strengthen the statistical power when exploring relationships within the data.

7. DATA MANAGEMENT

A selection of the results from the examinations will be presented to the participating subjects using a web portal developed by DaraLabs. This portal will also be used to enter or upload study data. The recording, storage and distribution of data are further explained in section 7.1.

7.1. Recording of data

The investigator will ensure that all data collected in the study, are recorded in a timely manner according to any instructions provided. Study data will be entered into a central database at the study site through an electronic system hosted by DaraLabs. The database will be hosted in a secure professional hosting facility with audited appropriate physical and logical security levels for the stored data.

In case of database failure, the data may be recorded on a paper based source data document. Study data will be entered by study staff. The electronic questionnaires, to be completed by the subjects will be distributed through the portal. The questionnaire will be available for completion by the subject in advance before the study visits (except for visit 1) and the subjects are encouraged to complete the questionnaire before the visits. If it has not been completed at the time for the visit,

the subject will have time to complete it on site. All system users, study personnel and study subjects, will have unique login details.

Results from the lab analysis will be uploaded to the database, and a selection of the results will be presented to subjects. The selection will be; length, weight, BMI, waist, hip, bio impedance (% fat), blood pressure, Hb, glucose, HbA1c, triglycerides, cholesterol, LDL, HDL.

The following safety measures will be taken to secure data quality:

- The electronic questionnaires, to be completed on an internet connected device (computer, tablet or smartphone) by study subjects, will be designed to make it as easy as possible for the subject to reply to all questions.
- At the completion of the study, the presence and distribution of all variables will be checked and compared intra-individually to detect any extreme outliers or obvious errors. Questionnaire data entered by the subjects themselves will not be analyzed/cleaned/excluded from the database through any similar procedure.

When these procedures are completed, a meeting with data management, a statistician and study management will be held. Questions on any data exclusions or corrections will be formally decided upon, clean file will be declared and the database locked. The reason for excluding any data declared as erroneous will be described in detail in the report from the meeting. The principal investigator is responsible for ensuring that a final clean file is declared and documented.

Data from The T2D Profile trial will be merged with data from SCAPIS or IGT Microbiota baseline examination.

7.1.1. Source data

Source data could be medical records, working sheets or be entered directly into the study database. The source will be defined in a source data document.

7.2. Data storage and distribution

The study organization at Sahlgrenska University Hospital responsible for interactions with participants in the study will use electronic case report forms (eCRF, Daralabs) for study data documentation. No PIN number are used, subjects are identified by their study code.

Direct analyses of blood via Clinical Chemistry at Sahlgrenska University Hospital are done within the hospital and PIN number are therefore used. However, data is transferred to eCRF and thereafter only identified by study code. Biobanking is done via the Sahlgrenska Biobank according standard routines within the hospital. Blood samples withdrawn from the biobank are labelled with study code only.

To directly interact with study participants a dedicated WEB-based application has been developed by DaraLabs AB. In this system, the study organization can communicate with participants and remind them of study visits and ask them to respond to questionnaires and give feed-back. The

participants will also receive feed-back on selected study data via the application as describe above.

At the SciLifeLab, data will be stored at the KTH/School of Biotechnology in pass word protected systems physically held at the Albanova (KYH) (Stockholm). Data will be analyzed and therefore also held at Department of Biology and Biological Engineering, Chalmers, Gothenburg (Prof. Jens Nielsens grupp).

Data generated within the study can be provided also to partners outside the project´s research team. Data can also be used in conjunction with partners in industry and companies. Data will be stored coded so identity cannot be revealed. The storage system will follow national guidelines for patient security and integrity for all data.

8. QUALITY CONTROL AND QUALITY ASSURANCE

8.1. Monitoring

All informed consents will be checked and the identity of the subject who signed the consent form will be verified.

8.2. Qualifications

All investigations will be performed by dedicated study staff. Study staff should be qualified to perform delegated tasks and be trained in study procedures.

9. STUDY FOLLOW-UP USING NATIONAL POPULATION REGISTRIES

No registry data will be used in this study.

10. CLINICAL FOLLOW-UP

The general recommendations for clinical follow-up are as follows:

- All subjects will be offered T2D management by a specialist in diabetology, as described in section 4.8.
- Immediate findings such as elevated plasma glucose levels and blood pressure elevation will be communicated to the subjects by the study nurse or study physician during a study visit and appropriate action according to hospital guidelines will be taken.
- A sample of the results from the lab analysis will be presented to the subjects in an individual website (see 7.1).
- Subjects who have isolated risk factors or deviating blood samples will be informed and advice given according to accepted guidelines.
- Changes in risk factors will be communicated to the participants by the study doctor / nurse using personal contact with the subjects using phone or letter.
- At the completion of the study each participant will receive a written summary of results from direct biochemical analyses and physical measurements.
- No action will be taken on results generated in the omics part of the study. This information is given in the Informed Consent Form (ICF) ICF.

11. ETHICS

11.1. Informed consent

The Principal Investigator at the center will ensure that the subject is given full and adequate oral and written information about the nature, purpose and possible risks and benefits of the study. Subjects must also be notified that they are free to discontinue from the study at any time. The subject should be given the opportunity to ask questions and allowed time to consider the information provided.

The subject's signed and dated informed consent must be obtained before conducting any procedure specifically for the study. The original, signed ICF must be stored in the Investigator's Study File. A copy of the signed ICF must be given to the subject.

If a protocol amendment requires a change to the ICF, the EC must approve modifications that lead to a revised ICF before the revised form is used.

11.2. Ethics committee

Te final study protocol, including the final version of the ICF a written approval must be approved or given a favorable opinion in writing by the Ethics Committee (EC) in Gothenburg before enrolment of any subject. The principal investigator is responsible for informing the Ethics Committee of any modifications and amendments to the protocol as per local requirement. The EC must also be notified by the Sponsor or designee in writing of the interruption and/or completion of the study.

11.3. Insurances

Subjects participating in The Molecular Profile of T2D trial are managed within the health care system and thus covered by the Patient Insurance according to the Swedish Patient Injury Act.

11.4. Ethical considerations

There are no physical dangers involved in any of the procedures. We will ensure that study participants receive appropriate T2D management according to guidelines and this treatment will not be any different from what would be offered in routine healthcare. Participants will be informed on clinical data collected at study visits as this may help the participant to improve his/her risk profile.

The integrity of the subjects needs to be secured. Relevant clinical information is documented in the Sahlgrenska University Hospital electronic patient journal system (Melior). All research data are used in coded format and in secure environments. We will in most cases publish data at group level to avoid identification of individuals. However, we will most likely want to publish data also from individual cases. In this case vital data will be changed so that individuals cannot be recognized.

One ethical issue in this study is the theoretical possibility that a subject harbors a major (highly penetrant) disease-causing mutation that may be revealed by whole genome sequencing. However

the likelihood of any previously unrecognized major disease-causing mutation is low in this age-group. Also, we will not apply bioinformatics strategies to actively search for clinically significant information. Furthermore, a disease causing variant should only be communicated after it has been verified (i.e. Sanger sequencing in an accredited clinical genetics laboratory) which we do not plan to do in this study. Therefore subject will be informed (orally and in the ICF) that no genetic information will be returned in this study. Likewise, participants will not be informed on other omics data since the clinical value is not known.

12. PROJECT ORGANISATION AND INFRASTRUCTURE

12.1. Stakeholders and Executive joint committee

The T2D Profile trial is led by an executive joint committee comprising representatives from two stakeholders: SCAPIS – Gothenburg (PI Göran Bergström, Sahlgrenska) and SciLIfeLab (KTH Center for applied proteomics – KCAP). Each stakeholder nominates one person (and one suppleant) to the executive committee. The executive joint committee has the overall scientific and fiscal responsibility for The T2D Profile study.

12.2. Report and publications

Results will be published in per-reviewed scientific journals.

13. STUDY TIMETABLE

The Molecular Profile of T2D Study is planned to start Q2 2016 in Gothenburg. The recruitment period is estimated to be 12 months and follow-up time 3 months. The study is expected to be completed by Q3 2017.

14. LIST OF REFERENCES

Ayoglu B, Haggmark A, Khademi M, Olsson T, Uhlen M, Schwenk JM, Nilsson P. Autoantibody profiling in multiple sclerosis using arrays of human protein fragments. Mol Cell Proteomics 2013; 12: 2657-72.

Chen R, Mias GI, Li-Pook-Than J, et al. Personal omics profiling reveals dynamic molecular and medical phenotypes. Cell 2012; 148: 1293-307.

Edsgärd D, Iglesias MJ, Reilly SJ, et al. GeneiASE: Detection of condition-dependent and static allele-specific expression from RNA-seq data without haplotype information. Sci Rep. 2016; 6: 21134.

Hood L, Price ND. Demystifying disease, democratizing health care. Science translational medicine 2014; 6: 225ed5.

Läkemedelsverket (Medical Products Agency). Läkemedelsboken 2014.

Schwenk JM, Igel U, Neiman M, et al. Toward next generation plasma profiling via heat-induced epitope retrieval and array-based assays. Mol Cell Proteomics 2010; 9: 2497-507.

Socialstyrelsen. Causes of death 2011. Stockholm. 2012.

Socialstyrelsen. Nationella riktlinjer för diabetesvård 2015.

Sommer F, Bäckhed F. The gut microbiota - masters of host development and physiology. Nat Rev Microbiol 2013; 11: 227-38.
