# Supplementary material for: Next generation plasma proteome profiling to monitor health and disease
Source: Nat Commun. 2021 May 3;12:2493. doi: 10.1038/s41467-021-22767-z (PMC8093230; doi:10.1038/s41467-021-22767-z)
Supplement: Supplementary file 3 — Description of Additional Supplementary Files [file 41467_2021_22767_MOESM3_ESM.docx]

File Name: Supplementary Data 1

Description: Complete list of the 1472 targeted proteins with the annotation of human secretome.

File Name: Supplementary Data 2

Description: Description and summary of the clinical data for the wellness and T2D studies.

File Name: Supplementary Data 3

Description: Differentially expressed proteins in male and female samples based on the wellness study. Three-way balanced ANOVA for gender with age and visit as covariates. Multiple test corrections have been applied for *P*-values using Benjamini and Hochberg method.

File Name: Supplementary Data 4

Description: Intra- and inter-individual variability of all proteins in a two-year wellness study.

File Name: Supplementary Data 5

Description: Summary of the 341 protein quantitative trait loci (Linear regression model adjusted for age and sex at baseline). Multiple test corrections have been applied for *P*-values using Benjamini and Hochberg method.

File Name: Supplementary Data 6

Description: Complete list of significant mixed-effect modeling results. Mixed-effect modeling analysis for each protein and clinical measurement adjusted for gender, age and visit. Multiple test corrections have been applied for *P*-values using Benjamini and Hochberg method

File Name: Supplementary Data 7

Description: Complete list of the pairwise Pearson correlation values between all proteins and clinical measurements.

File Name: Supplementary Data 8

Description: Differentially expressed proteins identified at baseline in the non-obesity group in T2D and wellness cohorts. Two-way balanced ANOVA for study cohort with gender as covariate. Multiple test corrections have been applied for *P*-values using Benjamini and Hochberg method.

File Name: Supplementary Data 9

Description: Most significant proteins associated with metformin treatment response. Mixed-effect modeling analysis for drug response with gender as covariate.
