# Supplementary material for: Next generation plasma proteome profiling to monitor health and disease
Source: Nat Commun. 2021 May 3;12:2493. doi: 10.1038/s41467-021-22767-z (PMC8093230; doi:10.1038/s41467-021-22767-z)
Supplement: Supplementary file 13 — Reporting Summary [file 41467_2021_22767_MOESM13_ESM.pdf]

nature research

Corresponding author(s): Mathias Uhlén

Last updated by author(s): Mar 1, 2021

Reporting Summary

Nature Research wishes to improve the reproducibility of the work that we publish. This form provides structure for consistency and transparency in reporting. For further information on Nature Research policies, see our Editorial Policies and the Editorial Policy Checklist.

Statistics

For all statistical analyses, confirm that the following items are present in the figure legend, table legend, main text, or Methods section.

n/a	Confirmed	
☐	☒	The exact sample size (n) for each experimental group/condition, given as a discrete number and unit of measurement
☐	☒	A statement on whether measurements were taken from distinct samples or whether the same sample was measured repeatedly
☐	☒	The statistical test(s) used AND whether they are one- or two-sided<br>*Only common tests should be described solely by name; describe more complex techniques in the Methods section.*
☐	☒	A description of all covariates tested
☐	☒	A description of any assumptions or corrections, such as tests of normality and adjustment for multiple comparisons
☐	☒	A full description of the statistical parameters including central tendency (e.g. means) or other basic estimates (e.g. regression coefficient) AND variation (e.g. standard deviation) or associated estimates of uncertainty (e.g. confidence intervals)
☐	☒	For null hypothesis testing, the test statistic (e.g. F, t, r) with confidence intervals, effect sizes, degrees of freedom and P value noted<br>*Give P values as exact values whenever suitable.*
☒	☐	For Bayesian analysis, information on the choice of priors and Markov chain Monte Carlo settings
☐	☒	For hierarchical and complex designs, identification of the appropriate level for tests and full reporting of outcomes
☐	☒	Estimates of effect sizes (e.g. Cohen's d, Pearson's r), indicating how they were calculated

Our web collection on statistics for biologists contains articles on many of the points above.

Software and code

Policy information about availability of computer code

Data collection	WGS: Qubit (v2.0), MIP-Mutation Identification Pipeline (v4.0.18), BWAmem (v0.7.17), GATK (v3.6), Manta (v1.0.3), reference genome GRCh38.p7
Data analysis	Plink (v1.9) for genome-wide association analysis; R project for statistical computation; packages: umap, lme4, ggplot2, tidyverse, circlize, fsmb

For manuscripts utilizing custom algorithms or software that are central to the research but not yet described in published literature, software must be made available to editors and reviewers. We strongly encourage code deposition in a community repository (e.g. GitHub). See the Nature Research guidelines for submitting code & software for further information.

Data

Policy information about availability of data

All manuscripts must include a data availability statement. This statement should provide the following information, where applicable:

- Accession codes, unique identifiers, or web links for publicly available datasets
- A list of figures that have associated raw data
- A description of any restrictions on data availability

All summary statistics and association data are available in the supplementary material. The participant-level genotype and phenotype datasets of S3WP program, including genetic mutations, plasma protein profiling with both NGS and qPCR readout, clinical chemistry, anthropometric measurements and questionnaires, have been deposited with the Swedish National Data Service (https://snd.gu.se/sv/catalogue/study/preview/88efa94d-39b3-4a50-8b3b-87b1abedefd4, a data repository certified by Core Trust Seal). Due to patient consent and confidentiality agreements, the dataset can only be made available for validation purposes by contacting snd@snd.gu.se. Data access will be evaluated according to Swedish legislation. Data access for research related questions in the S3WP program can be made available by contacting the corresponding author. Source data are provided with this paper.

Field-specific reporting

Please select the one below that is the best fit for your research. If you are not sure, read the appropriate sections before making your selection.

- [x] Life sciences
- [ ] Behavioural & social sciences
- [ ] Ecological, evolutionary & environmental sciences

For a reference copy of the document with all sections, see nature.com/documents/nr-reporting-summary-flat.pdf

Life sciences study design

All studies must disclose on these points even when the disclosure is negative.

Sample size	No a priori power calculation was made. The sample size was simply decided based on availability of analysis capacity and each individual is analysed three times. The design included the largest possible longitudinal study cohorts for both healthy and T2D, given the high frequency of visits and the extensive analyses.
Data exclusions	Only subjects with full longitudinal data and complete series of plasma samples were included in the analysis. The number of included individuals per dataset has been stated.
Replication	All of the plasma samples measured by PEA-NGS were also analyzed using PEA-qPCR as replication. Most of the assays (~82%) were found to correlate well with a high cross-platform correlation >0.7. A pairwise correlation of all protein levels across the 372 samples also showed high concordance with a median Pearson correlation of 0.985.
Randomization	Samples were randomized and several bridging samples were included on each plate for normalization
Blinding	No blinding. The study aims to give a “knowledge-based map” of the protein levels in blood. To validate possible biomarkers for follow-up studies, we strongly recommend blinding.

Reporting for specific materials, systems and methods

We require information from authors about some types of materials, experimental systems and methods used in many studies. Here, indicate whether each material, system or method listed is relevant to your study. If you are not sure if a list item applies to your research, read the appropriate section before selecting a response.

Materials & experimental systems

n/a	Involved in the study	
[ ]	[x]	Antibodies
[x]	[ ]	Eukaryotic cell lines
[x]	[ ]	Palaeontology and archaeology
[x]	[ ]	Animals and other organisms
[ ]	[x]	Human research participants
[x]	[ ]	Clinical data
[x]	[ ]	Dual use research of concern

Methods

n/a	Involved in the study	
[x]	[ ]	ChIP-seq
[x]	[ ]	Flow cytometry
[x]	[ ]	MRI-based neuroimaging

Antibodies

Antibodies used	PEA probes are generated by the manufacturer (Olink) from two paired antibodies, either matched monoclonal antibodies (mAb), one polyclonal antibody (pAb) split in two or a mix of both (one mAb and one pAb). For probe generation, antibodies were resuspended to 2 mg/ml in PBS according to the concentration stated by the antibody manufacture. Ten µg antibody was used in the conjugation reaction generating the PEA probes used in Olink Explore.
Validation	The analytical performance of PEA is validated for each protein assay by the manufacturer, included in the library and performance data is made available at www.olink.com. In short, technical criteria include assessing sensitivity, dynamic range, specificity, precision, scalability, endogenous interference and detectability in both healthy and pathological plasma and serum samples.

Human research participants

Policy information about studies involving human research participants

Population characteristics	The wellness study (or S3WP program): The study consists of 76 healthy individuals recruited from the Swedish CArdioPulmonary bioImage Study (SCAPIS), including 36 females and 40 males between 50-65 years of age. Participants were recruited on the criteria of being clinically healthy and not taking any medication for cardiovascular or pulmonary disease.<br><br>The T2D study: The T2D study is an extension of the S3WP program with the aim to perform molecular phenotyping of T2D before and after diabetes treatment. The T2D study consists of 48 subjects at age between 50 and 65 years with no history of diabetes who were diagnosed during population-based screening examinations at the Sahlgrenska University Hospital, Gothenburg. The diagnosis of diabetes was based on the Swedish standard, corresponding to the American Diabetes Association standards.<br><br>Eligibility were assessed at the first visit in SCAPIS (Swedish CArdioPulmonary bioImage Study) in consecutive order, stratified for sex to achieve an equal distribution of men and women. At the second visit in SCAPIS, eligible candidates received information about the S3WP study and were asked by the study nurse about their interest in participating.
Recruitment	Participants were either recruited from SCAPIS, or from another population-based study (the Impaired Glucose Tolerance and Gut Microbiota Study) that was conducted in parallel with SCAPIS and with the same cardiometabolic phenotyping as SCAPIS. Eligible subjects who were diagnosed with T2D in any of these studies received information about the T2D study and were be asked by the study nurse about their interest in participating.
Ethics oversight	Both studies are approved by the Ethical Review Board of Göteborg, Sweden. The registration number is 407-15 for the wellness study and 448-16 for the T2D study.

Note that full information on the approval of the study protocol must also be provided in the manuscript.
